# Planktonic eukaryotes in the Chesapeake Bay: contrasting responses of abundant and rare taxa to estuarine gradients

**DOI:** 10.1128/spectrum.04048-23

**Published:** 2024-04-12

**Authors:** Hualong Wang, Feilong Liu, Min Wang, Yvan Bettarel, Yoanna Eissler, Feng Chen, Jinjun Kan

**Affiliations:** 1 College of Marine Life Sciences, Frontiers Science Center for Deep Ocean Multispheres and Earth System, and Key Lab of Polar Oceanography and Global Ocean Change, Ocean University of China, Qingdao, China; 2 ECOSYM (Ecologie des systèmes marins côtiers)- UMR 5119, Universite de Montpellier, Montpellier, France; 3 Laboratorio de Virología, Centro de Neurobiología y Fisiopatología Integrativa, Instituto de Química y Bioquímica, Facultad de Ciencias, Universidad de Valparaíso, Valparaíso, Chile; 4 Institute of Marine and Environmental Technology, University of Maryland Center for Environmental Science, Baltimore, Maryland, USA; 5 Microbiology Division, Stroud Water Research Center, Avondale, Arizona, USA; Connecticut Agricultural Experiment Station, New Haven, Connecticut, USA

**Keywords:** planktonic eukaryotes, spatiotemporal variations, abundant and rare taxa, harmful algal species, Chesapeake Bay

## Abstract

Phytoplankton are important drivers of aquatic ecosystem function and environmental health. Their community compositions and distributions are directly impacted by environmental processes and human activities, including in the largest estuary in North America, the Chesapeake Bay. It is crucial to uncover how planktonic eukaryotes play fundamental roles as primary producers and trophic links and sustain estuarine ecosystems. In this study, we investigated the detailed community structure and spatiotemporal variations of planktonic eukaryotes in the Chesapeake Bay across space and time for three consecutive years. A clear seasonal and spatial shift of total, abundant, and rare planktonic eukaryotes was evident, and the pattern recurred interannually. Multiple harmful algal species have been identified in the Bay with varied distribution patterns, such as *Karlodinium, Heterosigma akashiwo, Protoperidinium* sp., etc. Compared to abundant taxa, rare subcommunities were more sensitive to environmental disturbance in terms of richness, diversity, and distribution. The combined effects of temporal variation (13.3%), nutrient availability (10.0%), and spatial gradients (8.8%) structured the distribution of eukaryotic microbial communities in the Bay. Similar spatiotemporal patterns between planktonic prokaryotes and eukaryotes suggest common mechanisms of adjustment, replacement, and species interaction for planktonic microbiomes under strong estuarine gradients. To our best knowledge, this work represents the first systematic study on planktonic eukaryotes in the Bay. A comprehensive view of the distribution of planktonic microbiomes and their interactions with environmental processes is critical in understanding the underlying microbial mechanisms involved in maintaining the stability, function, and environmental health of estuarine ecosystems.

**IMPORTANCE:**

Deep sequencing analysis of planktonic eukaryotes in the Chesapeake Bay reveals high community diversity with many newly recognized phytoplankton taxa. The Chesapeake Bay planktonic eukaryotes show distinct seasonal and spatial variability, with recurring annual patterns of total, abundant, and rare groups. Rare taxa mainly contribute to eukaryotic diversity compared to abundant groups, and they are more sensitive to spatiotemporal variations and environmental filtering. Temporal variations, nutrient availability, and spatial gradients significantly affect the distribution of eukaryotic microbial communities. Similar spatiotemporal patterns in prokaryotes and eukaryotes suggest common mechanisms of adjustment, substitution, and species interactions in planktonic microbiomes under strong estuarine gradients. Interannually recurring patterns demonstrate that diverse eukaryotic taxa have well adapted to the estuarine environment with a long residence time. Further investigations of how human activities impact estuarine planktonic eukaryotes are critical in understanding their essential ecosystem roles and in maintaining environmental safety and public health.

## INTRODUCTION

In aquatic ecosystems, planktonic eukaryotes play fundamental roles as primary producers, consumers, and decomposers in food webs (1, 2), and they also regulate biogeochemical cycles of various biogenic elements (3). It includes all unicellular eukaryotes (protists), fungi, and small zooplankton. Different groups of planktonic eukaryotes dominate or coexist in diverse habitats and play distinct ecological roles in balancing the ecosystem health (4, 5). For instance, photosynthetic protists, such as marine phytoplankton communities, produce 44–67 gigatonnes of fixed carbon per year, almost half of the total global net primary production (6, 7). Some phytoplankton (e.g., diatoms, dinoflagellates) form harmful algal blooms, and their global footprints can alter trophic interactions, and affect food webs, biodiversity, environmental and public health, and coastal socioeconomic development (8, 9). In addition, phagotrophic protists including ciliates and flagellates act as intermediate consumers and link primary and bacterial production to higher organisms (10, 11). Furthermore, planktonic fungi (mycoplankton), either in free-living filamentous and yeast forms or as parasites of other plankton (12), occupy a complex range of ecological niches such as saprotrophs, nutrient recyclers, and parasites (13). Given the broad genetic and physiological diversity of planktonic eukaryotes, exploring their composition, distribution patterns, and interactions with the environment is critical for understanding the ecological processes and mechanisms involved in maintaining ecosystem stability and function (14).

As the main boundary of land-ocean interaction zones, estuaries are among the most valuable, productive, and heavily used natural systems (15, 16). Human activities have significantly impacted the estuaries through nutrient enrichment, habitat loss, and changes in river flows and sediment export (17, 18). Seasonal and biological processes interact with anthropogenic activities to control the transport of water and sediments as well as biogeochemical transformations from freshwater to the ocean, so estuaries are ideal ecosystems for studying the impact of natural heterogeneity and human activities on aquatic planktonic eukaryotes (19). Chesapeake Bay is the largest estuary in the United States, and it supports the fishing industry valued of more than 3 billion dollars per year (20). To support these higher trophic consumers, microbial communities provide food sources (i.e., major primary production) and form the basis of aquatic food webs in the Bay. Therefore, studying the diversity and population dynamics of eukaryotic communities is also critical to maintaining the economic sustainability of the Chesapeake Bay.

Phytoplankton in the Chesapeake Bay have been studied since the mid-1970s, primarily based on microscopy and pigment analysis (21). More than 1,000 phytoplankton taxa have been identified in the Bay, including dominant groups such as diatoms, chlorophytes, cyanobacteria, cryptophytes, and dinoflagellates (22). A handful of toxin-producing species have also been recorded (22, 23). Many studies have focused on specific types of harmful algal blooms (HABs) and their relevance to natural environments in the Bay (24–28) as well as the responses to climate change (29). Satellite remote sensing (30, 31) and modeling of certain HAB species with their associated environmental conditions in the Bay have also been studied (32–37), such as *Pseudo-nitzschia* (38) and *Karlodinium veneficum* (39). Microscopy-based approaches are also widely used to investigate the composition of major phytoplankton groups like diatoms and dinoflagellates in the Bay. These microscopic approaches are suitable for larger plankton with distinguishable morphologies and can provide good visual observation and comparisons of plankton composition across samples. However, they are limited in distinguishing small plankton (i.e., smaller than a few microns) with less morphological features due to the lack of high-resolution identification (40, 41).

The use of molecular sequencing methods has found that small eukaryotes in the ocean are much more diverse than we learned based on the traditional approaches (42, 43). Advances in high-throughput sequencing have improved our ability to assess eukaryotic composition and population dynamics, as well as the potential to address their ecological impacts on an unprecedented scale in estuaries (19, 44–46). These detailed eukaryotic compositions are also helpful in identifying potential interactions (e.g., prey-predator) between microbiome populations and their relationships with causal environmental factors (47). Recently, we examined the Chesapeake Bay bacterioplankton community using high-throughput sequencing of 16S rRNA genes and found an interannually recurring pattern of bacterial community (48). The deep sequencing data also allowed us to study the potential relationships between prokaryotic populations in the Bay (49). As part of the microbial community, bacterioplankton are closely related to their eukaryotic counterparts (*a.k.a*. planktonic eukaryotes) mainly through food chains and niche interactions. Similar to bacterioplankton, planktonic eukaryotes including phytoplankton are highly sensitive and responsive to environmental variations and pollution. For instance, human activities and polluting inputs (e.g., herbicide residue) carried by run-off negatively affect the CO_2_ fixation and other critical ecological functions in coastal environments (50, 51). Planktonic eukaryotes are highly usable and critical for effect-based aquatic quality monitoring (52), especially in estuarine environments with strong human activities. However, to our knowledge, a detailed analysis of overall planktonic eukaryotes in the Chesapeake Bay is still missing. Up to date, a comprehensive view of the spatiotemporal dynamics of planktonic eukaryotes and their responses to environmental processes (e.g., nutrients, turbidity, and salinity gradient) in the Chesapeake Bay has not yet been defined.

In this study, we investigated the composition, diversity, and spatiotemporal distributions of planktonic eukaryotes (including total, abundant, and rare subcommunities) in the Chesapeake Bay using high-throughput sequencing analysis of 18S rRNA gene amplicons. The V4 region of the 18S rRNA gene, a conserved, universal marker gene but highly variable across the taxa, has been widely applied to investigate the diversity of microbial eukaryotes across the oceans (1, 53). A total of 73 surface water samples were collected at seven sites (along a spatial salinity gradient) across four seasons over three consecutive years. As the first interannual survey of total planktonic eukaryotes in the Chesapeake Bay, our results revealed noticeable diversity and distribution patterns under spatiotemporal variations. Distinct assembly of abundant and rare subcommunities affected by spatiotemporal variations and environmental filtering were observed in terms of richness, diversity, structure, and distribution. A stable and reoccurring annual distribution pattern for planktonic eukaryotes was also demonstrated in this study. Furthermore, potential environmental parameters contributing to the dynamics of planktonic eukaryotes in the Bay at both spatial and temporal scales were explored. Finally, potential bloom-forming phytoplankton groups and their spatiotemporal distribution throughout the Bay, such as diatom *Thalassionema*, *Pseudo-nitzschia*, *Rhizosolenids* and dinoflagellates *Alexandrium*, *Karlodinium veneficum*, and *Prorocentrum minimum* were also discussed.

## RESULTS

### Detailed structure and dynamics of planktonic eukaryotes in the Bay

Phytoplankton cells dominated the planktonic eukaryotes in the Bay. Counts and relative abundances of diatoms, dinoflagellates, Chlorophytes, and Cryptophytes to total phytoplankton cell counts were calculated and listed in Table S1. Among them, diatoms, dinoflagellates, and Cryptophytes were the main components, and their relative abundances ranged from 1.5% to 94.3%, 0.4% to 95.6%, and 0 to 90.0%, respectively. In general, dinoflagellates and Chlorophytes were more abundant in the cold season (winter and spring), while in the warm season (summer and autumn), diatoms and Cryptophytes dominated the phytoplankton community. Across the Bay, the distinct pattern was observed in winter: Dinoflagellates and Chlorophytes were abundant in the upper Bay, Cryptophytes in the middle Bay and diatoms in the lower Bay (Table S1). In summer, the overall counts of Chlorophytes were low across the Bay, and Cryptophytes were more detected in the middle Bay; both diatoms and dinoflagellates were widespread and abundant in the Bay but with no discernible distribution pattern. We compared the microscopic counts to the relative abundance of phytoplankton groups from the molecular assay (see details below), and significant positive associations were identified (*P* < 0.01) (Fig. S2), indicating a good agreement and consistency of these two independent methods.

**Fig 1:**
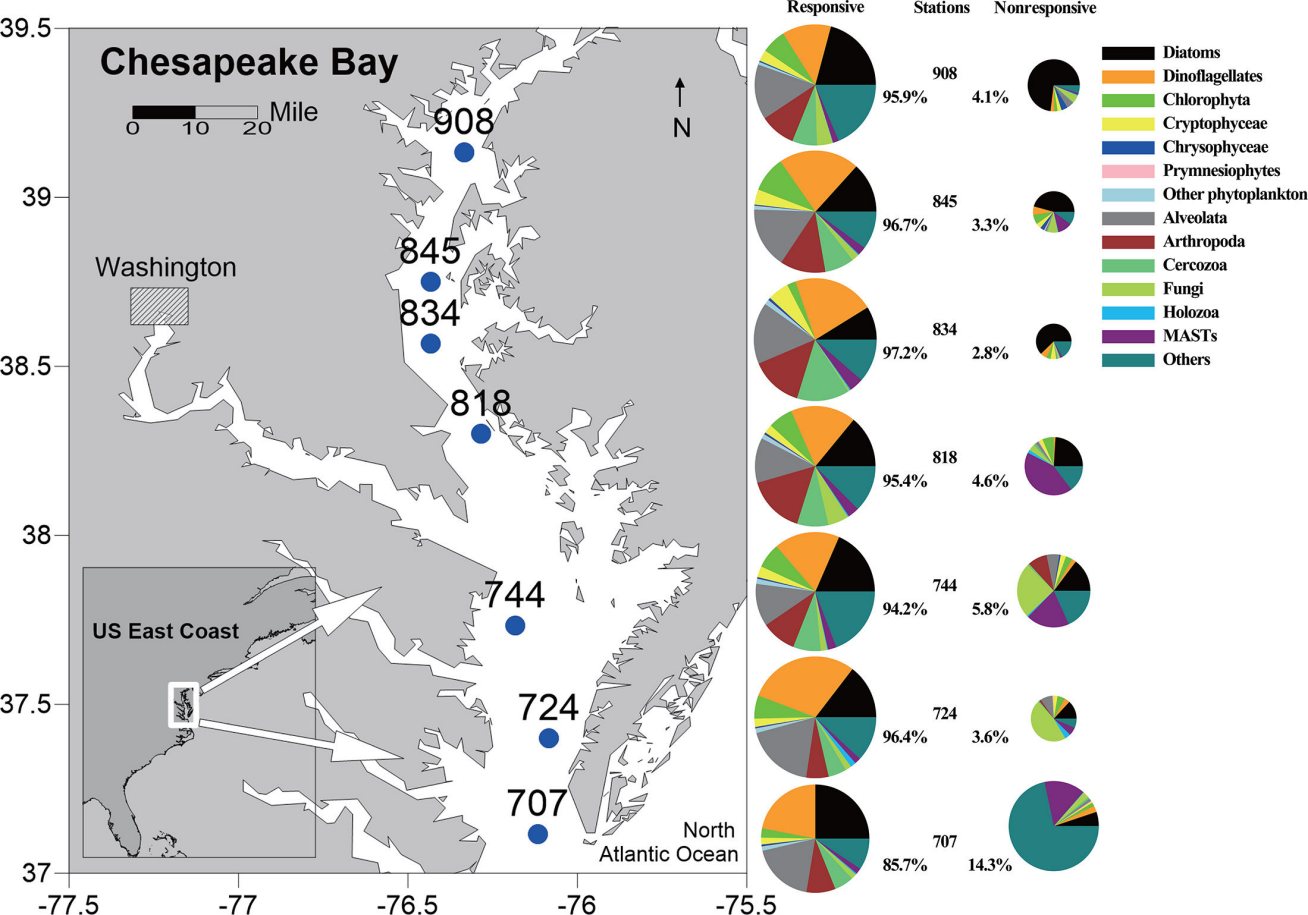
Map of the Chesapeake Bay showing sampling stations and the percentages of ASVs that are responsive/nonresponsive to environmental gradients. Pie size is proportional to the relative abundance of those ASVs that respond significantly (*P* < 0.01) (left) or not (right) to environmental factors at each site. Map was created using Ocean Data View version 5.4.0.

High-throughput sequencing results showed that eukaryotic microbial communities were dominated by phytoplankton (such as diatoms, dinoflagellates, Chlorophyta, Cryptophyceae, etc.), fungi, and marine stramenopiles (MASTs). The majority of the planktonic eukaryotes detected in this study responded to estuarine gradients (defined as responsive taxa in Fig. 1). The total relative abundances of responsive amplicon sequence variants (ASVs) ranged from 94.2% to 97.2% throughout the Bay, except the furthest downstream station 707 (85.7%) (Fig. 1).

**Fig 2:**
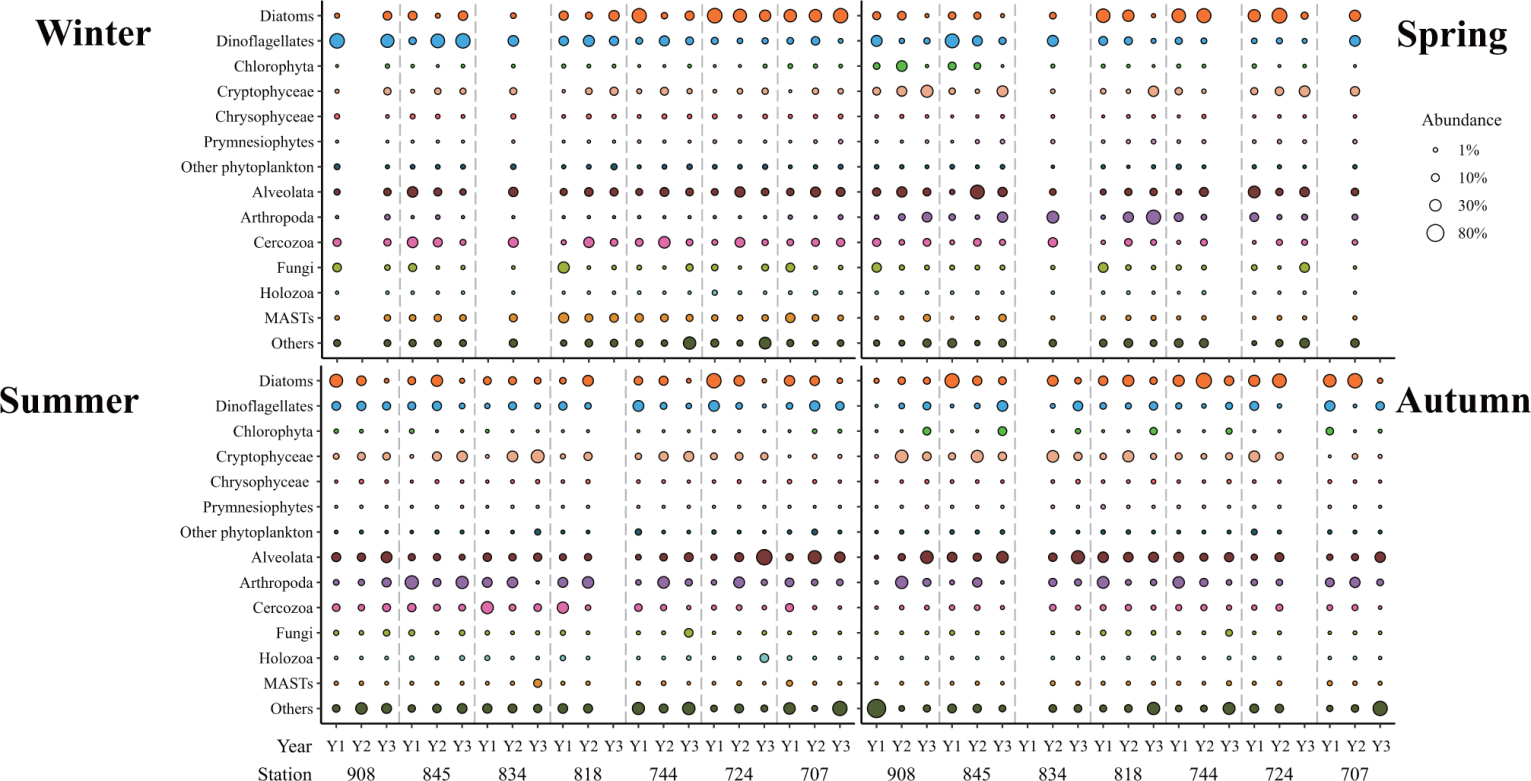
Relative abundance of major eukaryotic taxa in the Chesapeake Bay. Seasonal data (winter, spring, summer, and autumn) from each sampling site for three consecutive years (**Y1–Y3**) are included (unless it is unavailable). Bubble size represents the relative abundance of each taxon within each sample.

The distribution of major planktonic eukaryotic groups showed strong seasonal patterns (Fig. 2; Table S2). As we found in microscopic counts, diatoms were abundant throughout the year in all four seasons. Compared to spring and autumn, dinoflagellates, Cercozoa, and other phytoplankton were dominant in winter (*P* < 0.01), while Cryptophyceae were more common in autumn than in other seasons (*P* < 0.01). Relative abundances of Chlorophyta and Prymnesiophytes were significantly higher in spring than in other seasons (*P* < 0.01). Chrysophyceae and MASTs were more abundant in winter than in other seasons (*P* < 0.01), but Arthropoda displayed an opposite trend (*P* < 0.01). In general, dinoflagellates, Cercozoa, Chrysophyceae, MASTs, Cryptomycota, and other phytoplankton were negatively correlated with temperature (Table S3), although the total relative abundance of these macro-organisms (e.g., Annelida, Cnidaria, and Ctenophora) was lower than 2% across our collected samples in the Bay. On average, the relative abundance of phytoplankton communities increased from the middle Bay (station 818–834, 41.2%–42.0%) to the upper Bay (station 845–908, 46.2%–50.1%) and lower Bay (station 707–744, 46.5%–53.2%), and it was higher during spring (51.7%) and winter (51.4%) than summer (38.0%) and autumn (48.2%) (Table S4). The phytoplankton communities were negatively related to the increasing temperature and dissolved organic phosphorus (DOP) but positively related to the Chl *a* unsurprisingly (Table S3). As expected, subgroups within each phylum differed in seasonal patterns (Table S3). For instance, the relative abundances of MAST-1 and MAST-6 were negatively correlated with temperature, but MAST-3 and MAST-7 increased with temperature. Similarly, individual ASVs within each major eukaryotic group responded to temperature in different ways, such as those ASVs affiliated with dinoflagellata (305 ASVs), Chlorophyta (216 ASVs), diatom (216 ASVs), Chrysophyceae (109 ASVs), and Cryptophyceae (48 ASVs) (Table S4 and S5). For example, six ASVs of Chlorophyceae were positively correlated with the temperature while eight ASVs displayed an opposite trend. Seven ASVs of *Thalassiosira* were negatively related while two ASVs were positively correlated to the increasing temperature (Tables S4 and S5, *P* < 0.01).

Along salinity gradients, many major eukaryotic phyla exhibited distinct spatial variations in different seasons. The relative abundance of dinoflagellates decreased with increasing salinity gradients from the upper Bay to the lower Bay in winter but increased during spring (Fig. 2; Table S2, *P* < 0.01). Meantime, the abundance of Cryptophyceae was positively correlated with salinity gradients in winter, but not in other seasons. Cercozoa and fungi were negatively related to the increasing salinity during summer, and similar trends for Cercozoa and Holozoa were observed in spring. In general, the relative abundance of Cercozoa, Chytridiomycota, and Ciliophora decreased while Basidiomycota, Centrohelida, Cnidaria, and Haptophyta increased with the salinity gradients from the upper to lower Bay (Table S3, *P* < 0.01). Similar to the ASVs responding to temperature, individual ASVs within each eukaryotic group responded to salinity in different ways. For instance, five ASVs affiliated to *Thalassiosira* increased with higher salinity while three ASVs decreased with salinity in the Bay (Table S4 and S5, *P* < 0.01).

Spatiotemporal variations were found in the major families of microzooplankton in the Bay and detailed population structure across season and space were shown in Fig. S3 to S8. For example, the proportion of the family Oligotrichia (Alveolata) increased from the upper Bay to the lower Bay during winter, while the Calanoida (Arthropoda) was less common in winter (Fig. S3 and S4). The family Protaspidae (Cercozoa) and LKM11 (Fungi) were dominant in winter and spring (Fig. S5 and S6), and Acanthoecidae (Holozoa) and MAST 12A were more abundant in winter (Fig. S7 and S8).

Although many ASVs were significantly correlated with salinity and thus influenced by spatial gradients in the Bay, lots of ASVs were not affected by the salinity gradients. For example, the distribution of 30 out of 51 ASVs belonging to Chlorophyceae did not respond to spatial/salinity gradient. Some ASVs were specific to a location within a certain salinity spectrum: *Skeletonema* (ASV1168) was only abundant at station 908 while an ASV classified as *Neoceratium* spp. (ASV985, Dinoflagellata) dominated at stations 707 and 724 (Table S4). These results suggested that the long residence time in the Chesapeake Bay allows the adaptation of eukaryotic ASVs to different environmental niches in the Bay.

### Abundant and rare planktonic eukaryotes in the Bay

Rare subcommunities (2,386 ASVs) accounted for more than 86.5% of the total richness of eukaryotic microbes in the Bay, but their total relative abundance was only 11.3% of the overall community. Conversely, a rather low proportion of 5.9% ASVs (164 ASVs) was identified as abundant subcommunities, representing 77.1% of the entire eukaryotic community. The abundant subcommunities accounted for 82% of winter communities and 74% of summer communities, with rare subcommunities showing an opposite trend (winter, 9%; summer, 13%). The Venn diagram analysis showed that nearly all abundant taxa coexisted in four seasons, while most of the unique ASVs in each season were mainly from rare groups (Fig. S9). The number of unique ASVs in spring, summer, autumn, and winter was 161, 244, 85, and 150, respectively. In all, 584 ASVs were shared across the four seasons, with more than half of the shared ASVs belonging to the rare sub-groups. Interestingly, 628 ASVs were shared between summer and autumn, which was far more than shared ASVs between other seasons (Fig. S9).

Negative values of the mean nearest taxon distances in communities (SES.MNTD) indicated that phylogenetic clustering for these rare subgroups, that is, rare species within the community are more closely related than expected by chance (Fig. S10a). By contrast, positive values of SES.MNTD in the abundant subgroups indicated a higher phylogenetic evenness and was more distantly related than expected by chance. Furthermore, noticeably higher phylogenetic distances for rare eukaryotic microbial subcommunities were observed than those of the corresponding abundant subcommunities (Wilcoxon *P* < 0.001) (Fig. S10b). These results demonstrated that the distinct phylogeny of rare vs abundant microbial subcommunities reflects the evolution and niche adaptations of these groups, and subsequently implicates their differential sensitivities and responses to spatiotemporal changes in the Bay.

### Alpha diversity of planktonic eukaryotic communities

**Fig 3:**
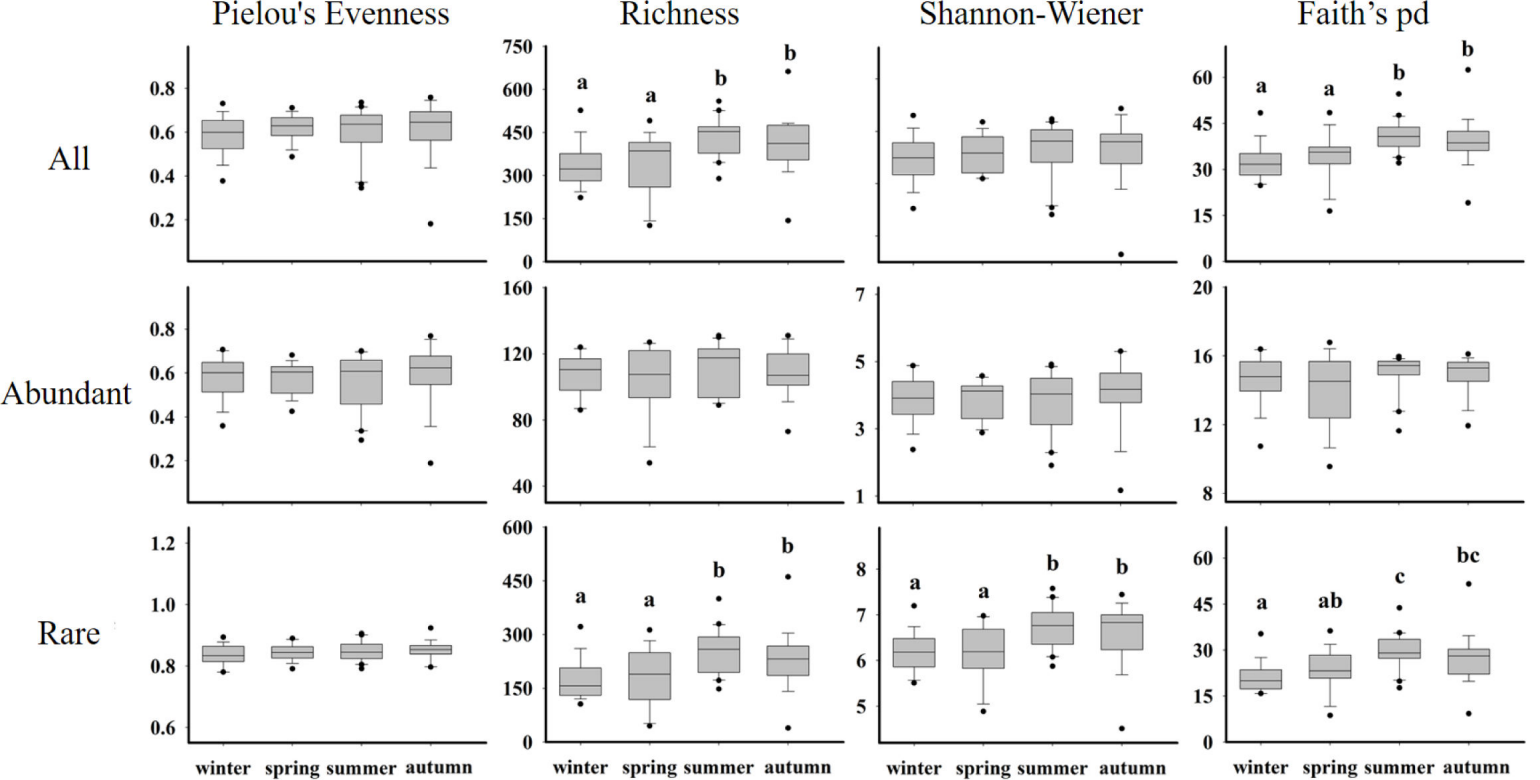
Diversity and richness of eukaryotic microorganisms (ASVs) across seasons in the Chesapeake Bay. Top, all taxa; middle, abundant taxa; and bottom, rare taxa. Different letters indicate significant differences between seasons.

Alpha diversity of eukaryotic communities (Shannon-Wiener, Faith’s-pd [phylogenetic diversity] and evenness indices) showed clear seasonal shifts in the Chesapeake Bay (Kruskal-Wallis, *P* < 0.01) (Fig. 3; Table. S6). Alpha-diversity indices in warm seasons were significantly higher than those in cold seasons (*P* < 0.01), although Pielou’s evenness and Shannon-Wiener index of total communities did not differ across seasons (Fig. 3). Rare eukaryotic planktonic microbes were major contributors to the total microbial diversity. Except for Pielous’s evenness, the diversity indices of rare subcommunities exhibited clear seasonal differences, but the abundant subcommunities did not differ between seasons (*P* < 0.01, Fig. 3; Table S6).

**Fig 4:**
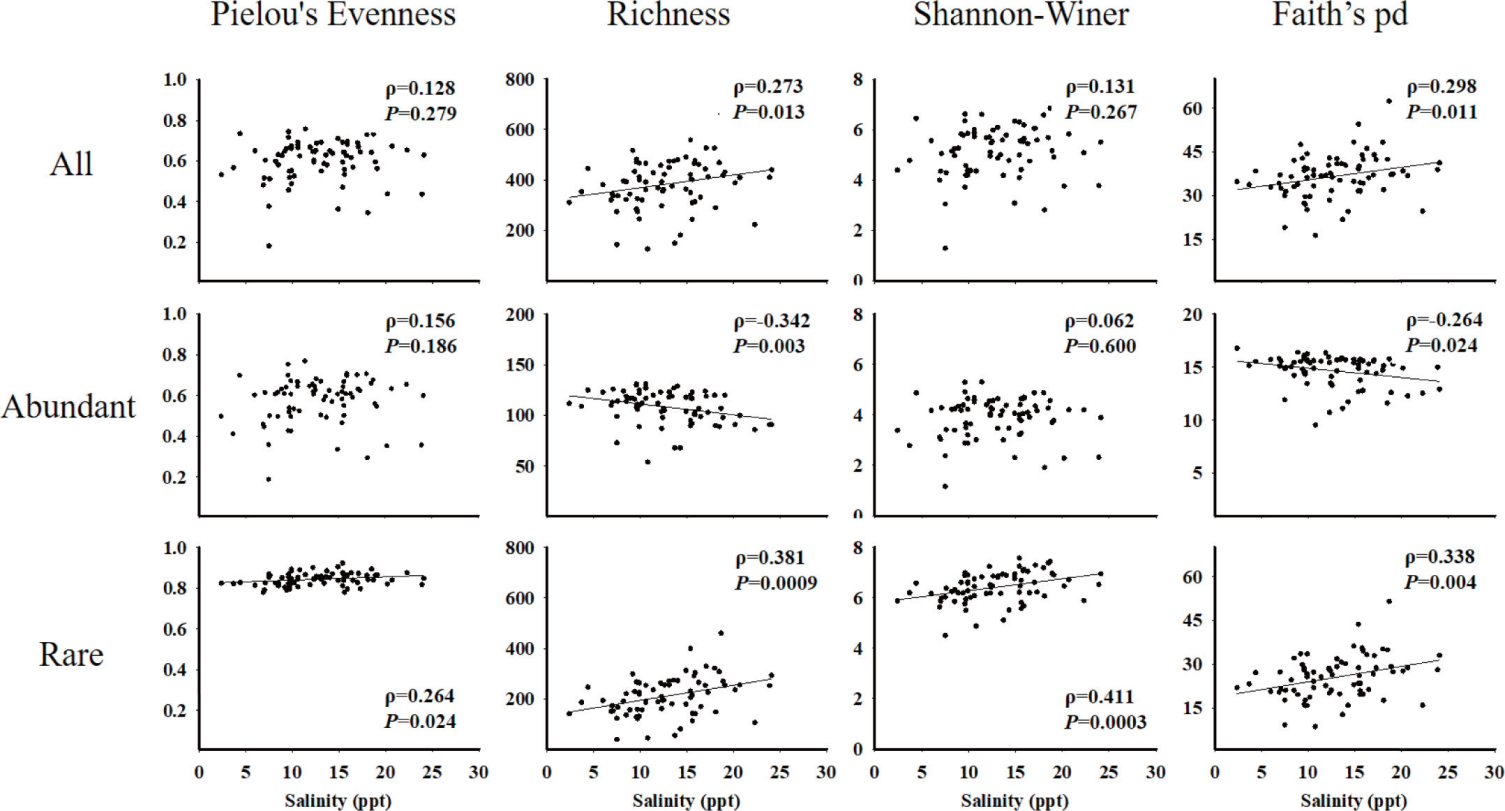
Diversity and richness of eukaryotic microorganisms (ASVs) along salinity gradients in the Chesapeake Bay.

When we compared the alpha diversity across spatial scales, variations along salinity gradients were also observed: the richness and phylogenetic diversity of the total planktonic eukaryotes were positively correlated with the salinity in the Bay (Fig. 4, *P* < 0.01). Further analysis showed that richness and Faith’s-pd indices of abundant and rare subgroups were both significantly correlated with the salinity gradient (*P* < 0.01). Interestingly, the diversity of rare groups was positively correlated with the salinity gradient, while the abundant groups were negatively related to the salinity gradient in the Bay. However, Pielou’s evenness and Shannon-Wiener indices of rare subgroups were significantly related to the salinity but the abundant subgroups were not. The rare communities were more sensitive to the salinity gradient and other environmental changes compared to the abundant groups (Fig. 4; Table S7). In summary, the richness and phylogenetic diversity indices of rare and total eukaryotic plankton communities responded positively to temperature, salinity, and turbidity, but negatively correlated with total nitrogen (TN), nitrate, and dissolved oxygen (DO). However, the abundant populations showed an opposite pattern (Table S7).

### Beta diversity of planktonic eukaryotic communities

**Fig 5:**
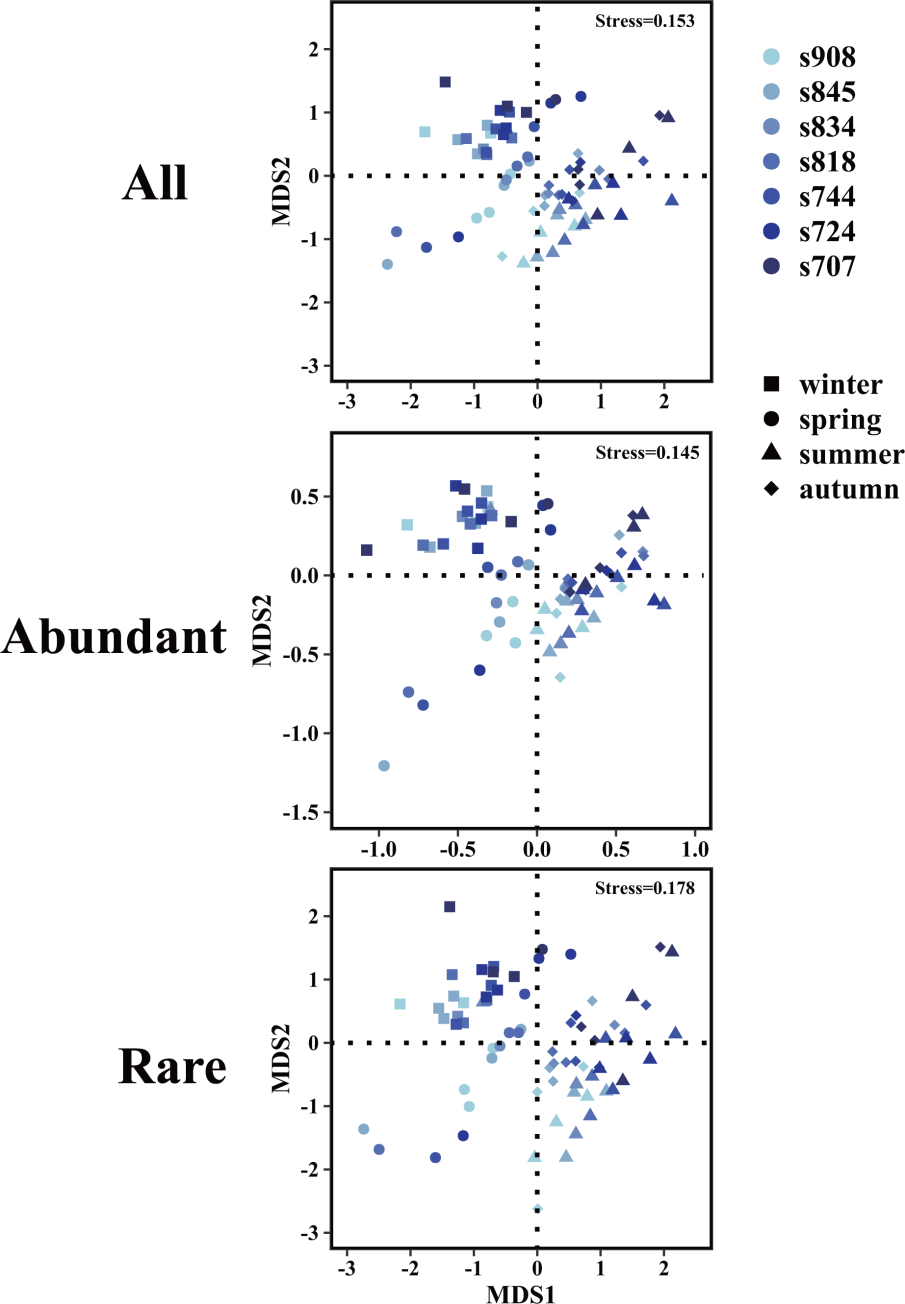
NMDS ordination plots for planktonic eukaryotes across season and space.

The non-metric multidimensional scaling (NMDS) plots displayed a clear seasonal shift and spatial variations of total, abundant, and rare eukaryotic communities in the Bay, and the distribution pattern of rare sub-communities was similar to the total community (Fig. 5). Thus, we infer the distribution pattern of total planktonic eukaryotes was mainly driven by rare sub-communities. Except for spring, the other three seasons were relatively stable and the summer-autumn communities were more similar between years (Fig. 5). Many phytoplankton species contributed to the variations in spring, and most of them were HABs-forming species. For example, one ASV belonging to Chlorophyta *Mychonastes* (ASV1892) in the year 2003 accounted for more than 30% of the total eukaryotic communities in the middle Bay, compared with less than 3% in other years (Table S4). In 2004, an ASV affiliated with diatom *Skeletonema* (ASV1168) dominated the eukaryotic community at station 908 (32.6%) but only accounted for lower than 0.6% at other stations and in other years (Table S4). The relative abundance of two Dinophyceae ASVs (ASV1035 and ASV 935) was higher than 20% at the lower Bay in 2004, while the proportion at other stations was lower than 0.5% in all 3 years (Table S4). These results also indicated that these species are more niche specific and an unique pattern of planktonic eukaryotes in spring.

The ANOSIM and Mantel tests revealed that seasonal variations, spatial shifts, and interannual differences occurred in planktonic eukaryotes in the Bay (*P* < 0.01) (Table 1). The smallest difference across seasons was identified between summer and autumn (R = 0.093), and the biggest discrepancy was obtained between winter and summer/autumn (R = 0.800/0.848). Similar patterns were observed in both abundant and rare sub-communities. However, the separation degree of rare sub-groups between cold and warm seasons was higher than those among spring vs summer/autumn as well as winter vs spring. Thus, winter planktonic eukaryotes were distinct from the other three seasons, and rare groups contained less differences between winter and spring compared to abundant groups. Interestingly, indicator ASVs of eukaryotic communities varied across seasons (Table S8), and the highest number with associated statistic values greater than 0.45 were found in winter (winter, 63; spring, 24; summer, 16; autumn, 30).

**TABLE 1:** ANOSIM and Mantel tests on comparisons of eukaryotic communities across season, year, and space (salinity) in the Chesapeake Bay^*a*^

Groups	All		Abundant		Rare	
	R	P	R	P	R	P
Seasonal						
All seasons	0.541	**0.0001**	0.522	**0.0001**	0.575	**0.0001**
Winter & spring vs summer & autumn	0.519	**0.0001**	0.494	**0.0001**	0.576	**0.0001**
Winter vs spring	0.675	**0.0001**	0.669	**0.0001**	0.552	**0.0001**
Summer vs autumn	0.093	**0.0064**	0.077	**0.0148**	0.210	**0.0001**
Winter vs summer	0.800	**0.0001**	0.789	**0.0001**	0.775	**0.0001**
Winter vs autumn	0.848	**0.0001**	0.837	**0.0001**	0.833	**0.0001**
Spring vs summer	0.322	**0.0001**	0.277	**0.0001**	0.528	**0.0001**
Spring vs autumn	0.477	**0.0001**	0.450	**0.0001**	0.555	**0.0001**
Inter-annual						
All years	0.114	**0.0003**	0.093	**0.0017**	0.175	**0.0001**
Year 2003 vs 2004	0.114	**0.0042**	0.100	**0.0072**	0.163	**0.0008**
Year 2003 vs 2005	0.170	**0.0010**	0.148	**0.0015**	0.227	**0.0001**
Year 2004 vs 2005	0.065	**0.0426**	0.037	0.1098	0.141	**0.0027**
Salinity						
Salinity in all samples	0.225	**0.0001**	0.217	**0.0001**	0.270	**0.0001**
Salinity in winter samples	0.626	**0.0001**	0.628	**0.0001**	0.562	**0.0002**
Salinity in spring samples	0.407	**0.0009**	0.437	**0.0003**	0.337	**0.0062**
Salinity in summer samples	0.462	**0.0003**	0.438	**0.0005**	0.550	**0.0001**
Salinity in autumn samples	0.538	**0.0001**	0.525	**0.0002**	0.622	**0.0001**
Temperature	0.544	**0.0001**	0.538	**0.0001**	0.467	**0.0001**
All stations (longitude, latitude)	0.132	**0.0003**	0.120	**0.0006**	0.190	**0.0001**

^*a*^ Significant results are in bold.

The inter-annual difference was also observed in the planktonic eukaryotes in the Bay, but not as obvious as those across seasons. Similar patterns occurred in both abundant and rare sub-communities (Table 1). Although not as strong as the seasonal impact, the Mantel tests demonstrated that spatial/salinity gradient had a strong influence on the distribution of eukaryotic community (Table 1). These spatial influences were more evident when the samples were analyzed based on each season, and less impact was detected in spring compared to other seasons (Table 1). In summary, our results showed that the total community, abundant, and rare subcommunity were all clustered strongly by season rather than the spatial gradients or inter-annual variation, and these variations were further demonstrated by rare groups than abundant ones (Table 1).

### Environmental processes affecting the eukaryotic plankton community

**Fig 6:**
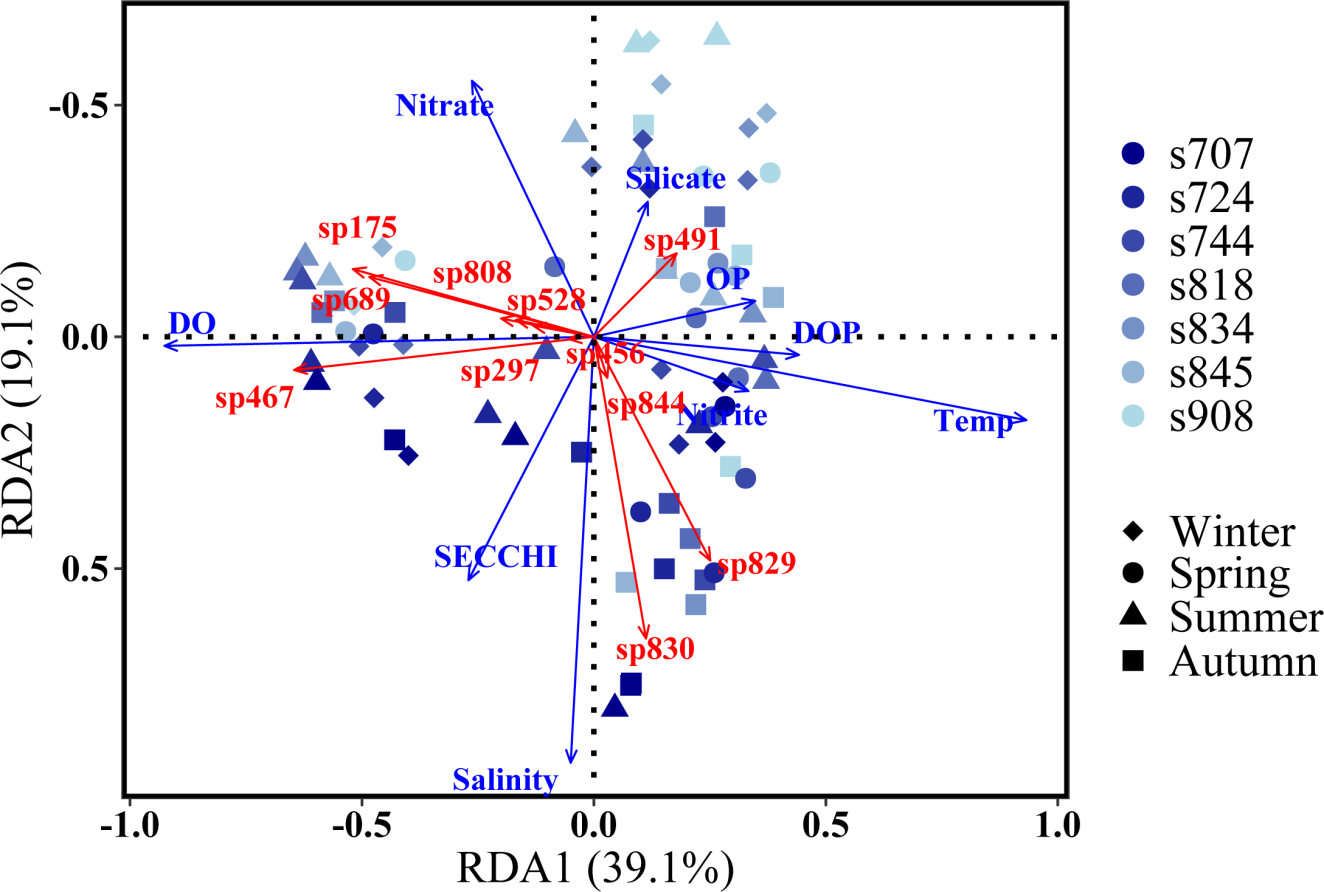
RDA ordination for the Chesapeake Bay planktonic eukaryotes in relation to environmental parameters. Only significant explanatory environmental factors (*P* < 0.05) (blue) and taxa (ASVs) with goodness-of-fit ≥0.38 (black) are included.

**Fig 7:**
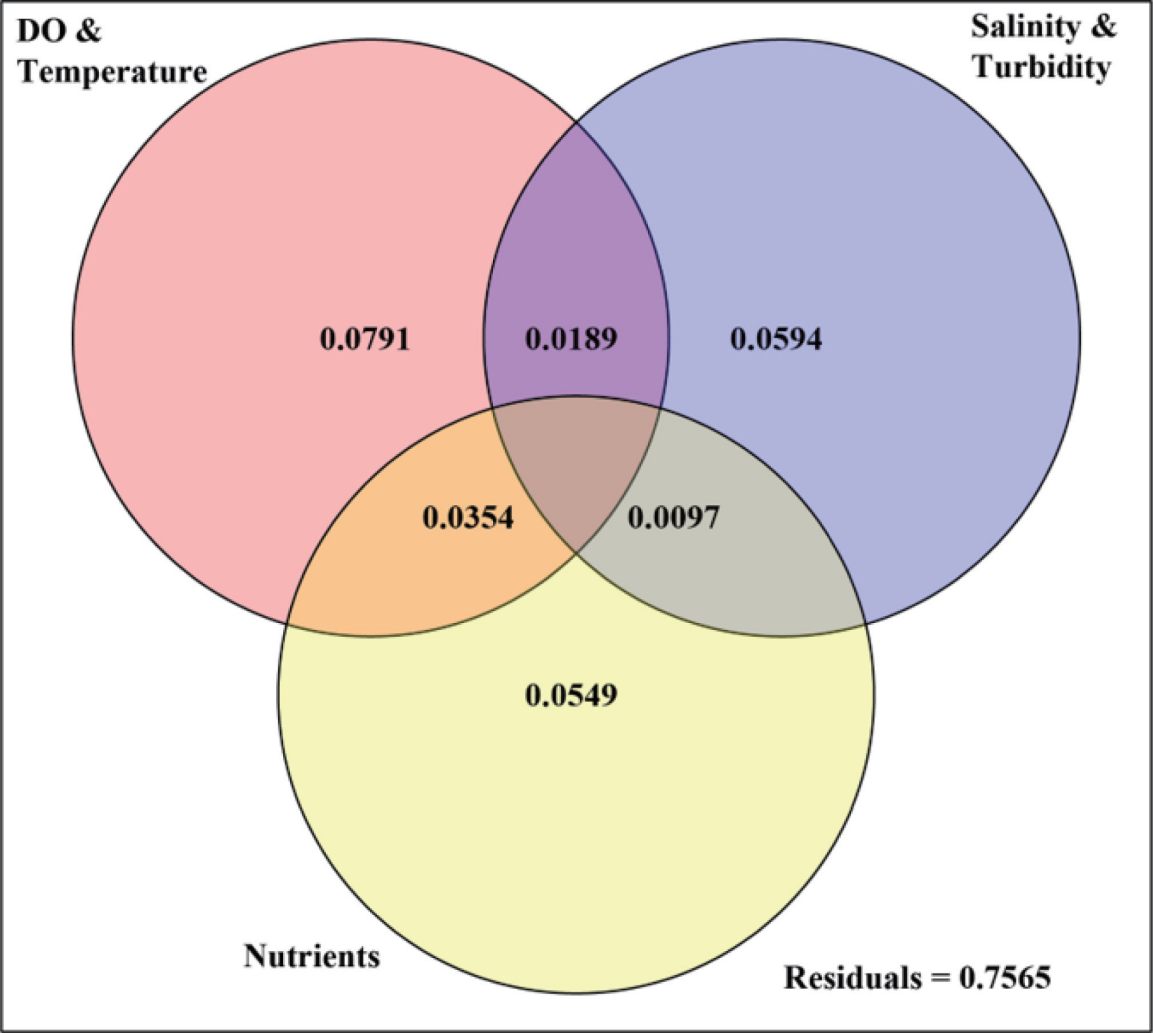
Variation partitioning analysis of eukaryotic communities and causing environmental factors in the Chesapeake Bay.

The variations of total community, abundant and rare subcommunity were significantly correlated with turbidity, TN, nitrate, dissolved oxygen (DO), and total phosphorus (TP) (*P* < 0.01) (Table S9). DOP, ammonium, phosphate, and total suspended solid (TSS) also accounted for the variations. The redundancy analysis (RDA) further confirmed these observations (Fig. 6). The horizontal axis (RDA1) explained 39.1% of the variability and the most contributing environmental variables were temperature, DO, DOP, organic phosphorus (OP), nitrite, turbidity, and nitrate. Samples from different seasons (i.e., winter, spring, and summer–autumn) were clearly separated by RDA1. The vertical axis (RDA2) separated samples spatially from the upper Bay to the lower Bay (with 19.1% of variations). Distribution of this spatial pattern on RDA2 was mostly explained by salinity, turbidity, nitrate, silicate and OP (Fig. 6). The variation partitioning showed that temporal variations (temperature and DO; 13.3%), nutrient availability (10.0%), and the spatial gradients (salinity and turbidity together; 8.8%) are the main environmental factors (Fig. 7). Environmental parameters were divided into three groups according to their significance and autocorrelations. For example, temperature was negatively related to the DO contents, and both of them changed with temporal (seasonal) variations. It is also noteworthy that the nutrient availability covaried with temporal variations and spatial gradients (Fig. 7).

The dynamics of most eukaryotic plankton groups/ASVs were able to be explained by combinations of explanatory variables (Fig. 6; Tables S3 and S5). For example, temperature, DOP, phosphate, nitrite, and silicate were negatively related to abundant taxa in winter [Diatom *Cerataulina* (sp467), Thecofilosea (sp175), MAST 6 (sp689), and Perkinsidae A31 (sp808)], but positively related to the occurrence of *Skeletonema menzellii* (sp491) and Syndiniales Group III (sp844) (Fig. 6). Two members of Syndiniales Group I (sp829, sp830) were more abundant in the lower Bay, where salinity was high and nitrate and silicate were low (Fig. 6). For clarity, only those taxa with high goodness-of-fit value (>0.38) were included in our RDA plot. Other eukaryotic plankton taxa that showed significant correlations with environmental parameters are listed in Table S5. Many taxa, even affiliated with the same genus from Diatom, Dinoflagellates, Chlorophyta, Cryptophyceae, and Chrysophyceae showed either positive or negative correspondence with different environmental factors (Table S5, *P* < 0.01). For instance, eight ASVs belonging to *Paraphysomonas* responded negatively to the salinity while the other two ASVs associated with *Paraphysomonas* correlated positively with salinity (Table S5, *P* < 0.01). Similar results were also observed in many other ASVs and genera with the corresponding environmental factors (Table S5).

Plentiful eukaryotic microbial populations were also significantly correlated with nutrient variation. For example, three members of *Thalassiosira* (ASV 1153, 1158, and 1160) were all positively correlated with nitrate concentration, and two members of them were also positively correlated with nitrite (Table S5). Furthermore, six members of Dinoflagellata *Paragymnodinium* (ASV 915, 916, 918, 919, 921, and 925) were negatively correlated with nitrate concentrations, while four of them were also correlated with nitrite concentration. These results showed that many eukaryotic microbial groups were potentially competitive to use/respond to inorganic nitrogen variations in the Bay, and detailed correspondences were included in the Supplemental Material (Tables S3 and S5). Similar results were also observed in regard of phosphorus sources. For example, four members of Chlorophyta Ulvophyceae (ASV 1749–1751, 1755) were all negatively correlated with DOP while two of them (ASV 1751 and 1755) were negatively correlated with phosphate concentration in the Bay. These groups could respond to the availability of phosphorus, including organic phosphorus, and utilize them as P sources. Furthermore, two members of Chlorophyta Trebouxiophyceae were significantly correlated with particulate carbon, and one of them was also related to turbidity and TSS in the Bay. Among these key environmental factors, salinity and turbidity play major roles in driving the spatial distribution pattern of the Bay eukaryotic microbial communities, especially those phytoplankton species, such as harmful algal species. For example, seven members of *Skeletonema* (ASV 1168, 1169, 1172–1175, and 1177) were positively correlated with silicate concentration while one strain of *Skeletonema* (ASV 1176) was negatively correlated with silicate concentration in the Bay. Thus, specific eukaryotic groups have distinct capabilities to adapt to different environmental gradients/habitats in the Bay, such as salinity, temperature, and nutrient availability.

## DISCUSSION

### Detailed eukaryotic communities and HAB species discovered in Chesapeake Bay

Deep sequencing analysis showed that ca. 2,800 eukaryotic ASVs were obtained in this study, and Chesapeake Bay contained high planktonic eukaryotic diversity with complex spatiotemporal dynamics. Our study included extensive samples collected from different seasons and locations over 3 years, representing a comprehensive and systematic survey of planktonic eukaryotes in the Bay. The community structure of planktonic eukaryotes or phytoplankton has been investigated and contributed a lot to our understanding and recognition of planktonic eukaryotes in the Bay, but most earlier studies were based on the traditional tools (i.e., microscopy or pigment analysis) or limited to sampling size (23, 54). Based on microscopy and continuous 20-year data with monthly sampling, H. G. Marshall et al. (24) characterized the phytoplankton composition and identified a total of 1,454 phytoplankton taxa in the Bay. Likewise, our microscopic counts identified that phytoplankton dominated our samples. Our results were also consistent with previous studies reporting that diatoms and dinoflagellates were the major phytoplankton groups along with significant contributions from other taxa such as chlorophytes and cryptophytes (22, 55). Compared to microscopy-based approaches, molecular techniques such as rRNA genes can provide higher resolution and help identify small plankton cells with fewer morphological features (40, 41). Despite PCR bias and variable copy numbers of 18S rRNA genes per cell (56), our high-throughput sequencing results revealed a distribution pattern of phytoplankton consistent with microscopic counts. Relative abundances of major phytoplankton groups from microscopic counts and molecular results correlated well (*P* < 0.01, Fig. S2), indicating consistency of microalgal distribution between the different methods. In addition, high-throughput sequencing provided detailed information and identities of other microeukaryotic groups besides phytoplankton. A few previous studies also applied this approach by investigating the microeukaryotic diversity in the oceans (2, 57). Therefore, the 18S rRNA gene sequencing of the debris or larvae of multicellular organisms could help us to understand their composition and distribution in the Bay along with other planktonic eukaryotes.

A total 216 diatom ASVs, 305 dinoflagellate ASVs, 216 chlorophyte ASVs, 109 Chrysophyceae ASVs, and 48 Cryptophyceae ASVs dominated the Chesapeake Bay phytoplankton communities. We also found many other phytoplankton groups occurred in the Bay, including Prymnesiophyceae (26 ASVs), Dictyochophyceae (36 ASVs), Eustigmatophyceae (10 ASVs), Raphidophyceae (9 ASVs), Pelagophyceae (5 ASVs), and Xanthophyceae (2 ASVs) (Table S4). Despite their relatively low abundance, these groups were also commonly found in other estuaries, such as the Baltic Sea (58), the Vistula River estuary (45), and sandy beaches (59). Characterization of the overall community structures *via* amplicon deep sequencing provides a comprehensive view of eukaryotic composition, diversity, and their spatiotemporal distributions in the Chesapeake Bay. For example, diatom (*Skeletonema* sp., ASV1168), dinoflagellata (Dinophyceae, ASV1033), chlorophyta (*Mychonastes*, ASV1892), and cryptophyceae (LKM11, ASV1408) were all abundant during spring (Table S5). These diverse eukaryotic phytoplankton communities were the main contributors to the primary production of the Bay, as well as the major players for CO_2_ fixation and nutrient biogeochemical cycling (60).

High-resolution sequencing also provided more detailed information on the composition and distribution of harmful algal bloom (HAB) species. Chesapeake Bay has a wide range of reported HABs, spanning almost all major genera (28, 31, 61–66). Almost 500 ASVs affiliated with the potential HAB species were identified in the Bay based on the database IOC-UNESCO Taxonomic Reference List of Harmful Micro Algae (HABs) (https://www.marinespecies.org/hab/). These species include but are not limited to diatoms *Thalassionema* (*T. nitzschioides* and *T. frauenfeldii*), *Pseudo-nitzschia* (*P. pungens* and *P. pseudodelicatissima*), five *Rhizosolenids* species, and dinoflagellates *Alexandrium*, *Karlodinium veneficum* (39), and *Prorocentrum minimum* (67, 68) (Table S4). One of our previous studies also identified a dinoflagellate bloom that occurred in early Spring at the Baltimore Inner Harbor, and the DNA sequencing facilitated the identification of multiple species that coexisted during the bloom (i.e., *Prorocentrum micans* and *Gymnodinum* spp.) (69).

Based on the high-throughput sequencing, this study identified extensive species and strains of HABs in the Bay with varied distribution patterns (as shown in Table S4). Among them, four ASVs were affiliated with Dinoflagellata *Karlodinium* (Table S4). *K. veneficum* is a HAB species found worldwide, and its bloom was first noted in the Chesapeake Bay in 1994 (70). *K. veneficum* produces Karlotoxin and has been implicated causing fish-kill events in the Bay (71) and failure of oyster spawning and development (72). *K. veneficum* blooms typically occur during summer and autumn in the Bay, which is likely associated with high P concentrations and lower dissolved inorganic nitrogen:phosphorus (DIN:DIP) ratios (28, 39). Our results also found that the ASVs affiliated with *K. veneficum* were positively correlated with environmental parameters such as temperature, salinity, Chl *a*, TN, total organic nitrogen (TON), dissolved organic nitrogen (DON), nitrate, nitrite, TP, OP, DOP, turbidity, PC, and silicate gradients (Table S5). There has been a trend of increasing bloom incidence and intensity since the mid-2000s, although fish kills remain sporadic as *K. veneficum* toxicity appears to be regulated by increased CO_2_ concentrations and P-limitation (73), water column stratification that alters salinity and nutrient flow regimes (74), and the abundance and nutritional status of cryptophyte prey (71).

It is worth mentioning that in addition to algal blooms, certain phytoplankton can produce toxins such as phycotoxin. The southern tributary region and coastal region of the Bay exhibited the highest amount of total phycotoxins while the phycotoxins reached their peak during the warm season (75). The distribution of phycotoxins is closely related to spatiotemporal variations of microbial communities in the Bay (75, 76). Phytoplankton can also concentrate toxic pollutants including herbicides, where intracellular atrazine concentrations are found 100 times higher than those in the environments (51). Similar bioaccumulations have been found in both freshwater diatom and green algae, and marine diatoms (77). This suggests that environmental pollution in coastal waters may be enriched to higher concentrations inside microalgal cells. More seriously, through bioconcentration and food chain transfer (e.g., zooplankton predation on microalgae), these toxin or pollutants can enter higher trophic levels and cause environmental or public health issues (77).

### Distinct patterns of abundant and rare eukaryotic plankton communities in the Bay

Our results identified abundant vs rare subcommunities based on their distinct structure and phylogenetic characteristics, which have been demonstrated in various environments such as European coastal waters (5), intertidal sediment environments (78), and wetland soils (79). The diversity of rare subcommunities in warm season was higher than cold season, but no difference was found in the diversity of abundant subcommunities across seasons. Almost all abundant ASVs were commonly found across four seasons, but unique ASVs in each season were mainly from rare groups. These results suggest that abundant planktonic eukaryotes are widely distributed in estuarine gradients and therefore are not environmentally constrained. By contrast, the rare subcommunities are more susceptible to environmental changes or ecological disturbances (80).

Lower phylogenetic distances for abundant subcommunities in the Bay indicated that closely related species exhibit similar ecological preferences across environmental gradients. Environmental variation across the Bay strengthens the estuarine ecological selection and increases the importance of deterministic shifts in the composition of the eukaryotic community because of the niche filtering (81). Due to the high abundance and wide distribution in the Bay, dominant microbes are less constrained by dispersal limitation. The abundant subcommunity with higher dispersal rates are intensified by drift (stochastic processes) or priority effects (82) so that the generalists are commonly present in the abundant subcommunity (83). Second, the abundant microbes have better environmental adaptation potential (79, 84), and therefore exhibited broader environmental thresholds to environmental factors (85) and/or broader species niche breadth (86). By contrast, rare taxa showed higher sensitivity to environmental changes with higher phylogenetic distances (as shown in Fig. S10). These results are in line with findings that rare taxa of bacterioplankton communities were more responsive to environmental factors than abundant taxa in the Yangtze River (China) spanning a distance of 4,300 km (87). Our results showed that rare and abundant eukaryotic microbial subcommunities possessed distinct phylogenetic characteristics and adaptations to spatiotemporal variations in the Chesapeake Bay.

The substantial number of eukaryotic microbial taxa found in the rare biosphere suggests that this subcommunity constitutes a diversity reservoir that can respond rapidly to environmental changes in the Bay (5). Certain eukaryotic microbial taxa (such as *Skeletonema* and Dinoflagellata *Neoceratium* spp.) in the Bay were abundant at one location but rare at others, suggesting their large oscillations in relative abundance. These results reflect strong fluctuations in planktonic eukaryotes across heterogeneous estuarine locations or seasonality at the same site (88). Rare taxa are hypothesized to include ecologically redundant taxa that could increase in abundance following environmental perturbations or changes, and maintain continuous ecosystem functions (89). Locally rare taxa can also serve as seeds for seasonal succession or spatial variations of the entire community in the Bay. Overall, our results indicate that, similar to subtropical estuaries (90), the Chesapeake Bay planktonic eukaryotes contain predominantly abundant and rare subcommunities with distinct structuring patterns and phylogenetic characteristics across spatial gradients and seasonal shifts. Different estuarine ecological driving forces govern the spatiotemporal shifts of abundant and rare microbial subcommunities, implying more complex mechanisms may be at play to shape both subgroups, and tremendous diversity of the rare subcommunities may be subjected to more complicated ecological processes (90). Further analyses of the functional aspects of these abundant and rare groups will contribute altogether to a better understanding of the community assembly and their links to estuarine ecosystem processes.

### Estuarine gradients drive the spatial and temporal variations of planktonic microbiomes in the Bay

Eukaryotic and prokaryotic microbial communities were affected by both temporal and spatial variations in the Bay. Variation partitioning analysis demonstrated that temporal (season), spatial (salinity) variations, and nutrient availability are the three major driving parameters in explaining the dynamics of planktonic eukaryotes (13.34%, 8.8%, and 10%) as well as prokaryotes [16.2%, 7.2%, and 15.7% (48)]. Higher temperatures likely result in earlier spring blooms, which could cause trophic decoupling or changes to the spatial distributions of particular prokaryotic and eukaryotic groups (91). The increase in the proportion of HABs species was observed during spring in the Bay, such as the dinoflagellata (Dinophyceae) and diatom (*Cylindrotheca closterium*). Higher temperatures can also affect microbial metabolic processes. For example, phytoplanktonic respiration increases with temperature more rapidly than photosynthesis (92).

In addition to temperature, nutrient availability is critical to temporal variations in primary production and biomass, and therefore restructure the microbial community composition in the Bay. Nutrient limitation leads to lower Chl *a* and a replacement with an increasingly diverse flora in the Bay (55). The increase in eukaryotic richness negatively related to the total nitrogen gradients in the Bay was observed in this study. On a global scale, total nitrogen input and phytoplankton production have a strong correlation in estuarine and marine waters (8). In addition, strong spatial and temporal heterogeneity in carbon cycling in response to riverine inflows were observed in the Bay: net heterotrophy prevailed in the upper bay and upstream tributaries while the middle and lower bay region displayed net autotrophy. The main stem of Chesapeake Bay is somewhat unique in its tendency toward net autotrophy as a consequence of its large size and extremely high nutrient to organic matter input ratio (93). Temporal variations in riverine organic matter and nutrient loads appear to greatly regulate the metabolism in the Bay (94). To date, the pattern of nutrients remains unchanged in the majority of the Bay, suggesting that nutrient loads should be further reduced in the Bay to achieve a less nutrient-saturated ecosystem (95, 96). Nutrient enrichment also contributes greatly to the development of large biomass blooms, leading to anoxia and even toxic or harmful impacts on fisheries resources, ecosystems, and human health or recreation (8).

Salinity has been shown to have a strong impact on shaping prokaryotic and eukaryotic microbial communities in the estuarine/coastal environments, such as the Baltic Sea (19). In our study, the diversity and distribution patterns of prokaryotic and eukaryotic microbial communities demonstrated distinct responses to the salinity continua in the Chesapeake Bay: the richness and Shannon-Wiener indices of prokaryotic microbial communities were negatively correlated with salinity while the ones of planktonic eukaryotes were positively correlated with salinity gradients [(48) and this study]. This agrees with the previous results that mixing with marine water led to quick changes in species diversity on the freshwater side in both the Chesapeake Bay and the Baltic Sea (97). Indeed long-term observations have shown that phytoplankton richness, composition, and function (i.e., resource use efficiency) can be predicted by salinity (97, 98). Along with the salinity gradients from freshwater to ocean, turbidity decreased and light became more available at the lower Bay. Light availability can affect the spatial distribution of certain microbial groups such as phototrophic microorganisms (both photoautotrophs and photoheterotrophs), such as Chlorophyta. For instance, the relative abundance of photoheterotrophic microbes (i.e., rhodopsin-producing microbes) increased from the upper Bay to the lower Bay (99). Altogether, our results suggest that estuarine spatial/salinity shifts contribute more in explaining the dynamics of eukaryotic microbial communities compared to prokaryotic communities [(48) and this study]. Different microbial groups adapt to distinct environmental gradients in the Bay, such as temperature, nutrient availability, and salinity (100, 101), and these environments have a pervasive effect on the phytoplankton biomass and primary productivity.

Under current projected scenarios of global-scale anthropogenic perturbation, nearly every critical control on an aquatic microbial community is currently in flux, such as temperature, irradiance, nutrient availability, environmental pollution, and toxicity (102). These future changes can lead to replacements of estuarine microbial species by taxa better suited to the changing environments, thereby altering beta-diversity and resulting in biotic homogenization or differentiation (103). For example, most microbial groups have a diversity minimum under stress environments and results in reduced biological diversity and interaction complexity in the coastal environments (97). In addition, environmental variations can reduce the original functional richness and shift the community to a new functional state (104). The loss in functional groups across the season and space in the Bay may lead to cascade effects where biotic homogenization degrades ecosystem processes such as nutrient cycling due to the shifts in phytoplankton functional composition (105). To mitigate these impacts, ecological preservation and restoration can be achieved by enhancing environmental heterogeneity and dispersal, consequently avoiding local-scale taxonomic and functional homogeneity in estuaries and therefore maintaining environmental and public health status (104).

## MATERIALS AND METHODS

### Sample characterization and high-throughput sequencing

In total, 73 surface water samples (500 mL, below 2 m) were collected at seven stations along the spatial gradients seasonally from 2003 to 2005 (10 samples for station 908, 845, 744; 6 samples for station 834; 11 samples for station 724; and 7 samples for station 707) (Fig. 1; Table S1). The samples were categorized seasonally based on water temperature (48, 106). Multiple years of monitoring in the Bay also indicated that water temperature is a good indicator of seasonal changes (48). The temperature could vary across months, such as May and June. Therefore, we divided the samples into four seasons as follows: winter (February and March) <10°C, summer (August) > 25°C, and the spring (May and June) and autumn (October) samples between them.

Sample collection and preparation procedure have been described previously (107). The collected water samples have prefiltered through a 200-micron mesh before further filtered through 0.2 µm Millipore polycarbonate filters, and filters are archived at −80°C for long-term storage. A small subsample (10 mL) was taken from all samples and fixed with Lugol’s fixative (at a final concentration of ~2%) for microalgae counting, and stored in the dark at 4°C. Based on their morphological features, microalgal groups (Diatom, Dinoflagellate, Chlorophyta, and Cryptophyceae) were examined and counted with Utermöhl Sedimentation Chambers using an inverted microscope (Olympus model IX51, Olympus Corp., Tokyo, Japan) (108). Temperature and salinity were measured during the sampling cruises. The other abiotic data were obtained from the Chesapeake Bay Program’s (CBP) Water Quality Database (https://www.chesapeakebay.net/what/downloads/cbp_water_quality_database_1984_present), including Chl *a*, DO, silicate, TN, ammonium, TON, DON, nitrate, nitrite, TP, DOP, orthophosphate phosphorus, and Secchi depth. The CBP stations are reasonably close to our sites (within 2–3 km), although they do not have the exact coordinates as our stations. Plenty of previous researches and our studies have shown clear and repeatable spatiotemporal patterns in the Bay (48, 65, 107), and thus the CBP data closest to our sampling dates and sites were applied in our analysis.

Environmental DNA extraction has been described in J. Kan et al. (107), and all DNA were preserved and archived at −80°C for long-term storage. DNA quantity was assessed by NanoDrop 2000 spectrophotometer (Thermo Fisher Scientific Inc., Waltham, MA). Library preparation and amplicon high-throughput sequencing followed the 18S Metagenomic Sequencing Library Preparation protocol from Illumina (https://support.illumina.com/). 18S rRNA genes were amplified using universal eukaryotic primers for the V4 region, namely 582F (5′-GCGGTAATTCCAGCTCCAA-3′) and 706R (5′-AATCCRAGAATTTCACCTCT-3′) (53). Studies have shown that short amplicon sequences of the V4 region can provide a diversity profile similar to that obtained from the entire 18S rRNA gene (109). Polymerase chain reaction contained 3 µL environmental DNA (20 ng µL^−1^) template, 25 µL 2 x Premix Taq, and 1 µL each primer (10 mmol L^−1^) in a volume of 50 µL, and were amplified with following thermocycling program: 5 min at 94°C for initialization; 30 cycles of 30 s denaturation at 94°C, 30 s annealing at 52°C, and 30 s extension at 72°C; followed by 10 min final elongation at 72°C. High-throughput sequencing of 18S rRNA genes was performed on an Illumina Nova6000 platform (paired-end 250 bp mode), following the manufacturer’s guidelines. Raw sequencing data used in this study are available through the GenBank database with BioProject number: PRJNA898840.

### Sequence analyses

The QIIME 2 software package (version 2021.2) was used to process the raw sequence data (110). In brief, a total of 10,594,000 reads were obtained from 73 samples after demultiplexing. q2-DADA2 was used for removing primers, denoising, filtering, merging, and chimera removal before generating ASVs. The reads were trimmed to the same length (forward at 230 bp and reverse at 230 bp), and in total of 8,165,889 clean reads were obtained. A Naïve Bayes classifier artifact (https://github.com/qiime2/q2-feature-classifier) was applied to assign the ASVs to taxa at 99% using the classifier data set (Silva 138 99% full-length sequences). An alpha rarefaction analysis at a sampling depth of 80,666 sequences was used to document that samples were sequenced to a sufficient depth (Fig. S1). After quality filtering and reads control, a total of 7,866,578 high-quality sequences were clustered into 2,730 ASVs for eukaryotic microbial communities. For all ASV-based analyses, the original ASV table was normalized/rarified to a depth of 80,666 sequences per sample to minimize the sampling effects. The QIIME 2 package was also used to generate Bray-Curtis distance matrices and α-diversity metrics including evenness, observed ASVs, phylogenetic diversity, and Shannon-Wiener diversity. We defined the rare and abundant taxa depending on the cutoff level of relative abundance (0.01% and 1%) across samples (80, 85). Briefly, the abundant taxa consisted of ASVs (i) with relative abundance ≥1% in at least one sample and (ii) with average abundance ≥0.1% across all samples. The rare taxa composed of ASVs with a relative abundance <0.01% in all samples, and ASVs with a relative abundance <0.01% in some samples but never ≥1% in any sample. The remaining ASVs were grouped as the “intermediate” taxa.

### Statistical analyses

Statistical analyses were mainly completed using R statistical software (version 4.1.0). Differences between major eukaryotic microbial groups across seasons were compared using one-way ANOVA (*P* = 0.01). Tukey’s *post hoc* tests were used to test the hypothesized differences, which ensured that the differences were qualified, and the statistically significant results (*P* ≤ 0.01) were identified. The relative abundance of 18S rRNA genes that significantly correlated to environmental gradients (based on correlations) was classified as responsive, and no significant relationships as non-responsive (Fig. 1). Differences in the eukaryotic microbial community diversity (alpha and beta diversity) were determined using Kruskal-Wallis test (alpha diversity) and PERMANOVA (beta diversity) through QIIME2 (version 2021.2) (110). NMDS was performed based on the Bray-Curtis distance matrices to demonstrate variations in eukaryotic microbial communities over spatial and seasonal scales. To estimate the phylogenetic clustering of rare and abundant eukaryotic microbial taxa in the Bay, we calculated a standardized index using the mean nearest taxon distance (SES.MNTD) using the “ses.mntd” function in the “picante” package (111). To evaluate the pairwise phylogenetic distance across samples, the beta mean nearest taxon distance (βMNTD) was calculated using the “bmntd” function in the “picante” package. Analysis of similarity (ANOSIM) was used to compare eukaryotic microbial communities between groups across spatial and temporal scales. The influence of continuous environmental variables such as salinity gradients on eukaryotic microbial distribution was also calculated with the Mantel test. The NMDS, ANOSIM, and Mantel analyses were performed using “metaMDS,” “anosim,” and “mantel” functions in the “vegan” R package. Relationships between environmental conditions and the relative abundance of major groups/taxa were tested using Spearman’s rank correlation coefficient with the “corrplot” R package. Only taxa representing at least 0.5% of total number of sequences were used for correlation analysis.

RDA of the vegan R package was used to explore the relationships between environmental parameters and the eukaryotic microbial communities. This analysis was calculated from Hellinger-transformed eukaryotic microbial ASV count data and the original environmental variable data (112). The explanatory variables with strong linear dependencies/correlations (variance inflation factors > 10) were excluded before the analysis (112). To identify the “best” explanatory variables in turn and decide when to stop the selection, forward selection with function “ordistep” in the “vegan” package was used. The taxa with goodness-of-fit at least 0.4 in the ordination plane formed by axes 1 and 2 were shown in the RDA plot. The significance of the RDA model was tested by ANOVA based on 999 permutations. This analysis could help to determine the most influential factors and to what extent these environmental parameters affected microbial composition. In addition, variation partitioning analysis was performed using the “vegan” package to quantify the variations explained by subsets of environmental factors, such as temperature and DO, salinity and turbidity, and nutrient availability.

## Data Availability

The 18S rRNA gene sequence data have been submitted to the NCBI SRA database under the accession number SRX18233088-SRX18233160. Additional processed data sets are available upon request from the authors.

## SUPPLEMENTAL MATERIAL

Supplemental figures spectrum.04048-23-s0001.docx Fig. S1 to S10; Supplemental Table legends.

Supplemental tables spectrum.04048-23-s0002.xlsx Tables S1 to S9.

ASM does not own the copyrights to Supplemental Material that may be linked to, or accessed through, an article. The authors have granted ASM a non-exclusive, world-wide license to publish the Supplemental Material files. Please contact the corresponding author directly for reuse.
