# Supplementary material for: Planktonic eukaryotes in the Chesapeake Bay: contrasting responses of abundant and rare taxa to estuarine gradients
Source: Microbiol Spectr. 2024 Apr 12;12(5):e04048-23. doi: 10.1128/spectrum.04048-23 (PMC11064499; doi:10.1128/spectrum.04048-23)
Supplement: Supplemental figures — Fig. S1 to S10; Supplemental Table legends. [file spectrum.04048-23-s0001.docx]

**Planktonic eukaryotes in the Chesapeake Bay: Contrasting responses of abundant and rare taxa to estuarine gradients**

**Hualong Wang ^1^, Feilong Liu ^1^, Min Wang ^1^, Yvan Bettarel ^2^, Yoanna Eissler ^3^,** **Feng Chen ^4,^ * and** **Jinjun Kan ^5,^ ***

^1^ College of Marine Life Sciences, Frontiers Science Center for Deep Ocean Multispheres and Earth System, and Key Lab of Polar Oceanography and Global Ocean Change, Ocean University of China, Qingdao, China

^2^ ECOSYM (Ecologie des systèmes marins côtiers)- UMR 5119, Universite de Montpellier, Montpellier, France

^3^ ﻿Laboratorio de Virología, Centro de Neurobiología y Fisiopatología Integrativa, Instituto de Química y Bioquímica, Facultad de Ciencias, Universidad de Valparaíso, Valparaíso 2360102, Chile

^4^ Institute of Marine and Environmental Technology, University of Maryland Center for Environmental Science, Baltimore, USA

^5^ Microbiology Division, Stroud Water Research Center, Avondale, USA

*Address correspondence to Jinjun Kan, [jkan@stroudcenter.org](mailto:jkan@stroudcenter.org) or Feng Chen, [chenf@umces.edu](mailto:chenf@umces.edu).


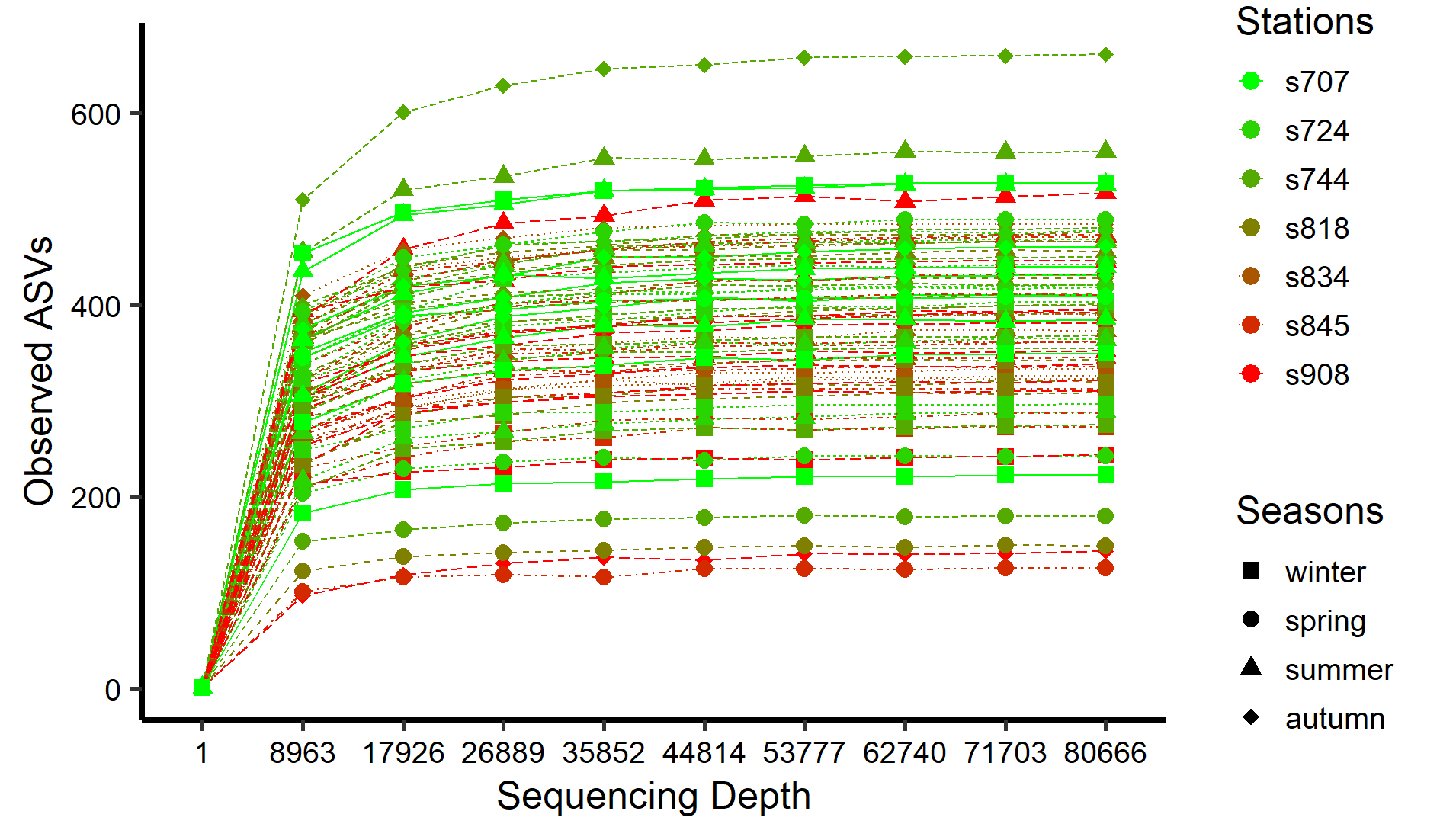


**FIG S1.** Rarefaction curves that calculated at the normalized subsample size of 80,666 sequences per sample, showing the effect of sequencing depth on observed number of ASVs in our samples.


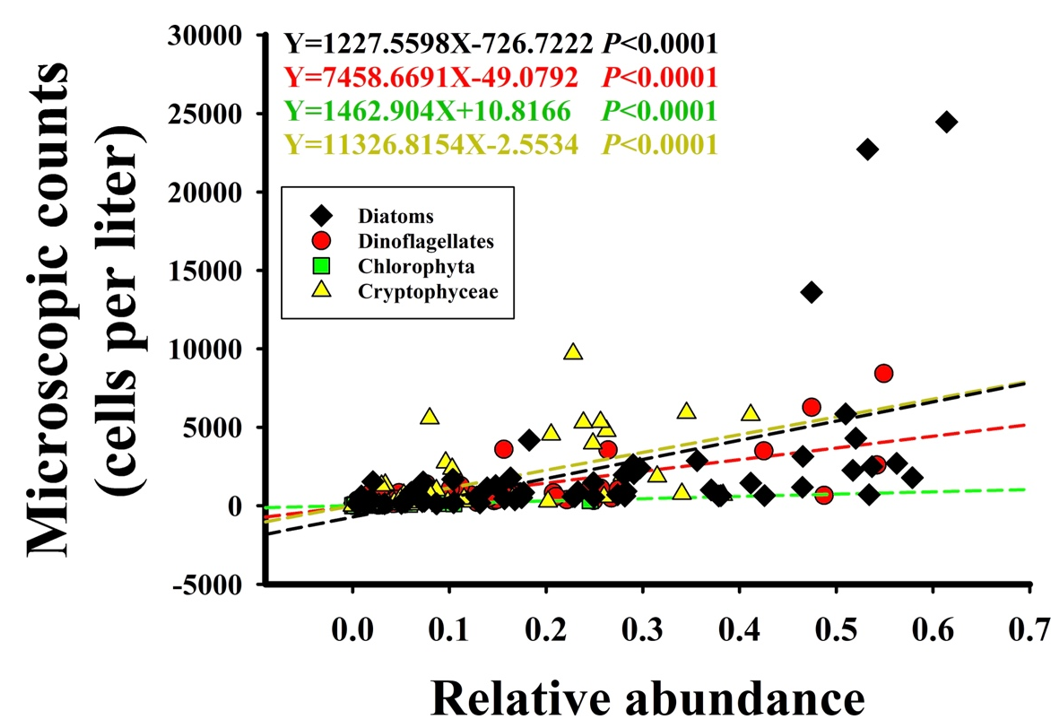


**FIG S2.** Correlations between cell counts and high-throughput sequences (relative abundance) of major microalgal groups in the Chesapeake Bay.

**
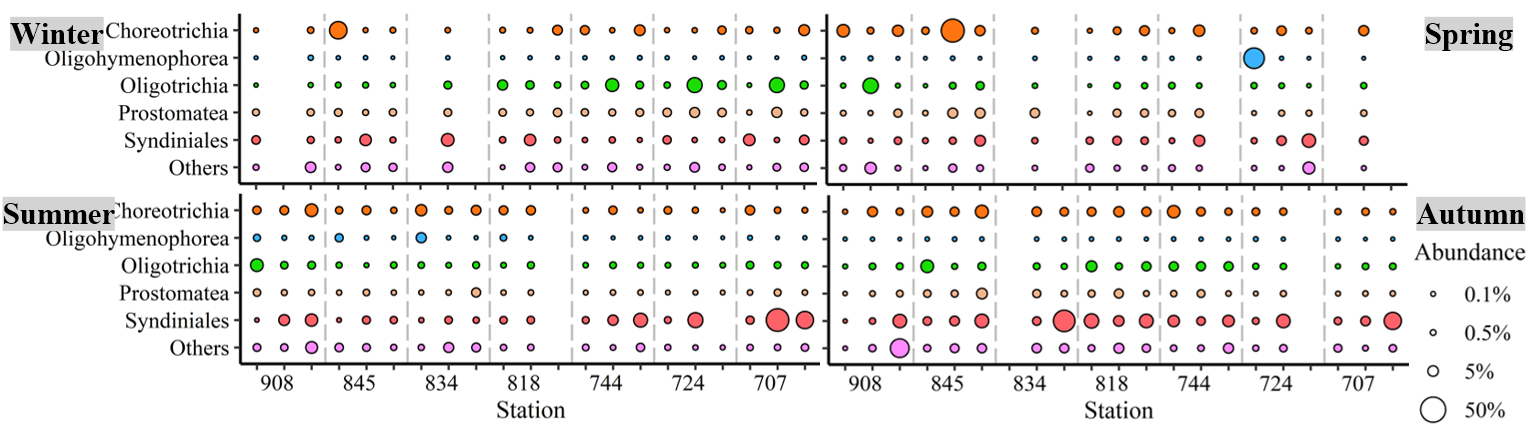
FIG S3.** Relative abundance of major families of Alveolata in the Chesapeake Bay. Seasonal variations (winter, spring, summer and autumn) from each sampling site for three consecutive years are included (unless it is unavailable). Bubble size represents the relative abundance of major families within each sample.

**
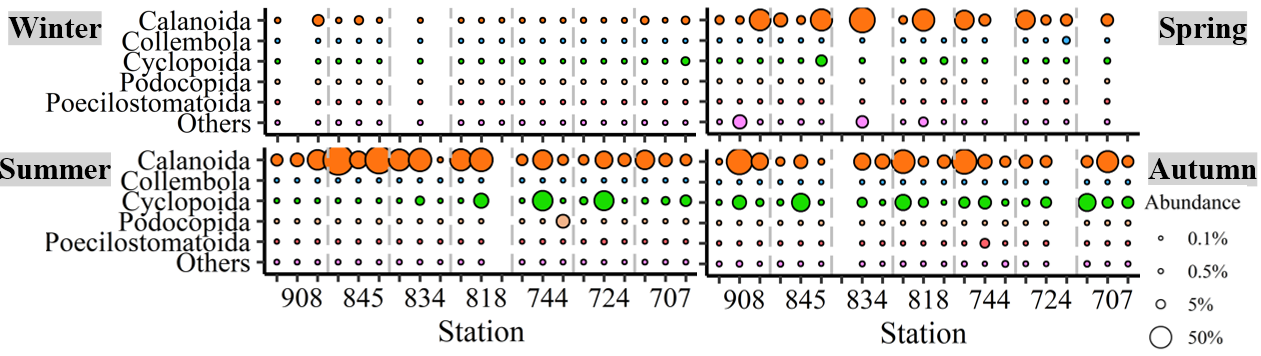
**

**FIG S4.** Relative abundance of major families of Arthropoda in the Chesapeake Bay. Seasonal variations (winter, spring, summer and autumn) from each sampling site for three consecutive years are included (unless it is unavailable). Bubble size represents the relative abundance of major families within each sample.

**
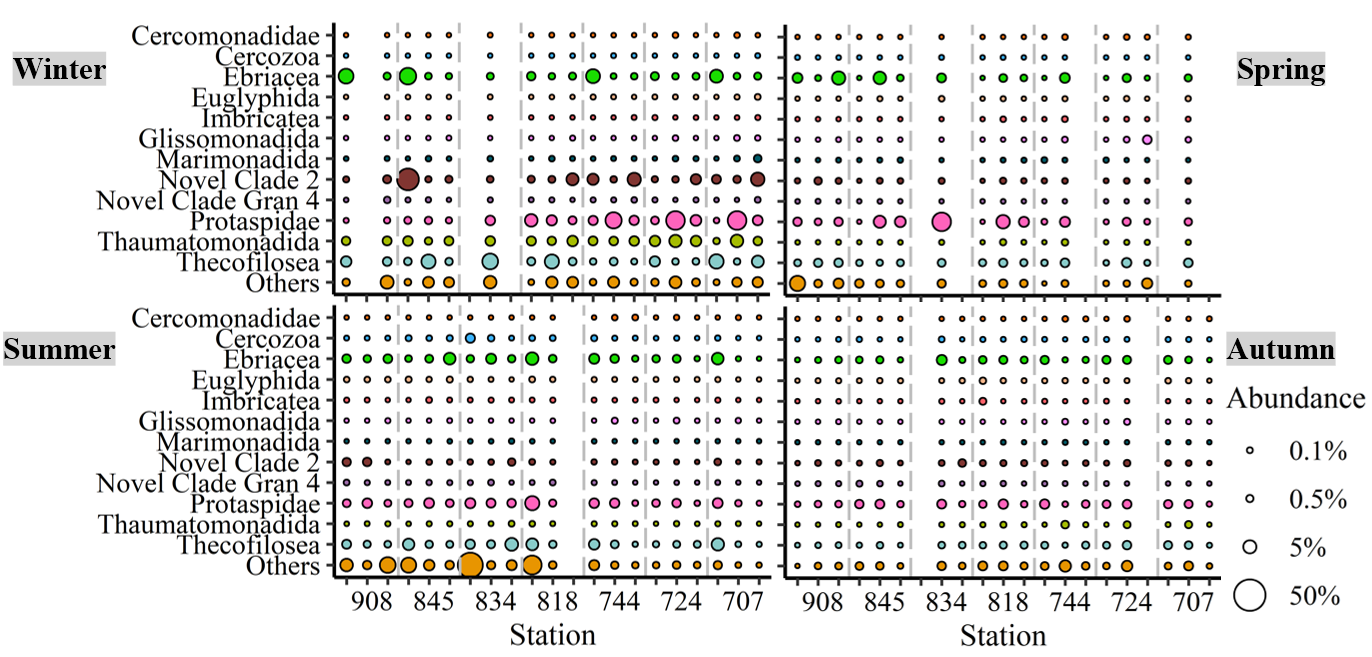
**

**FIG S5.** Relative abundance of major families of Cercozoa in the Chesapeake Bay. Seasonal variations (winter, spring, summer and autumn) from each sampling site for three consecutive years are included (unless it is unavailable). Bubble size represents the relative abundance of major families within each sample.


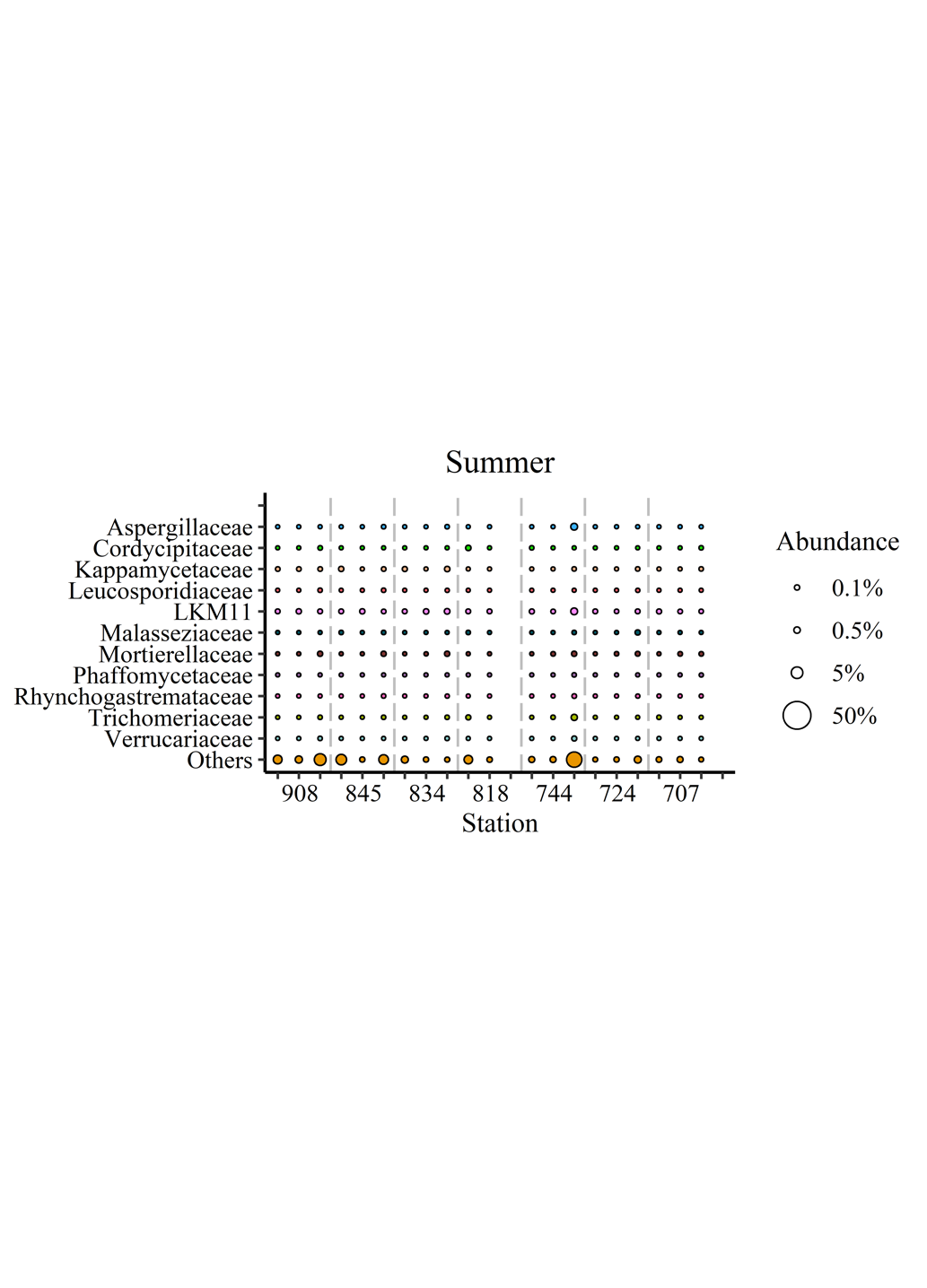

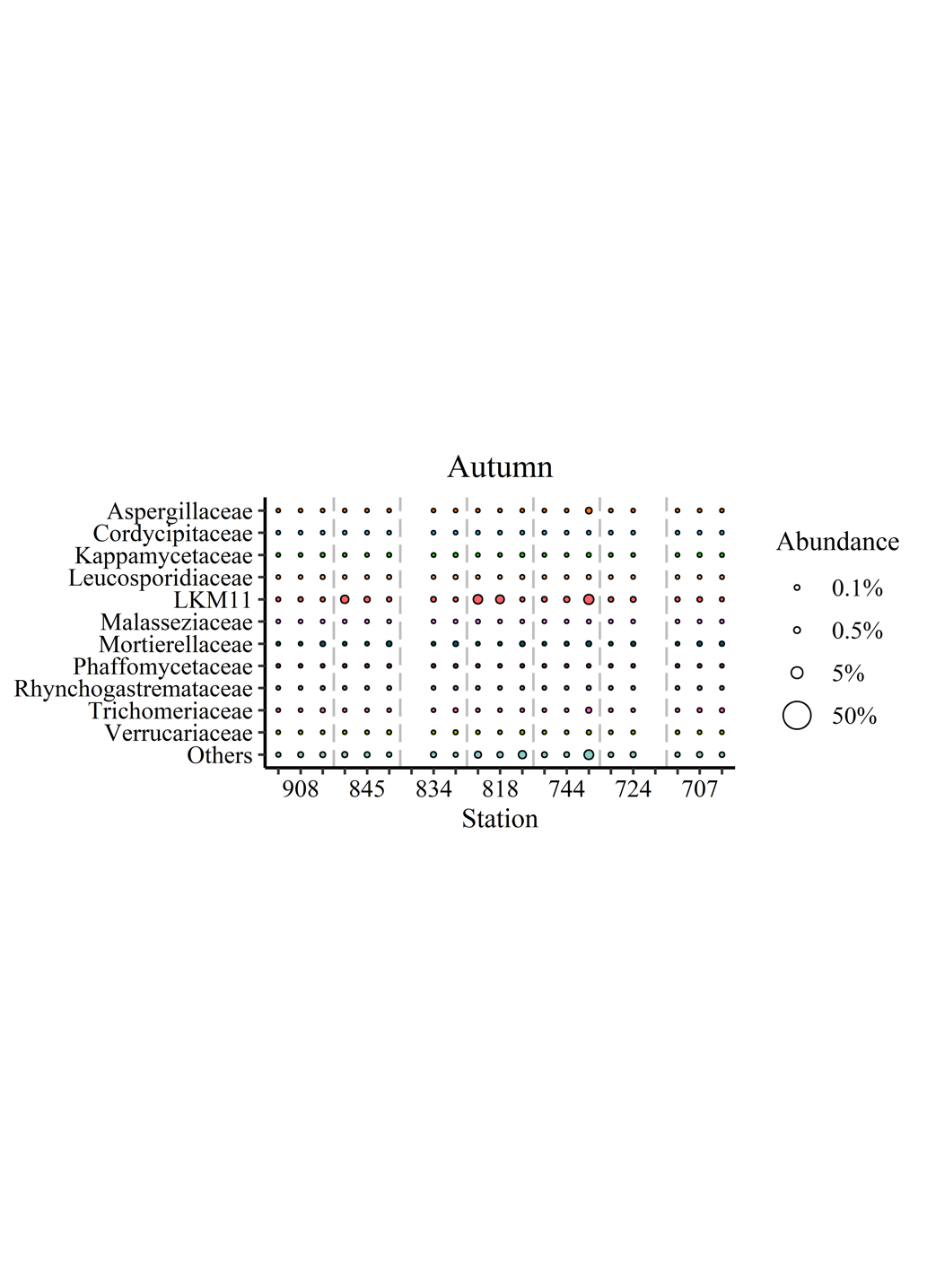

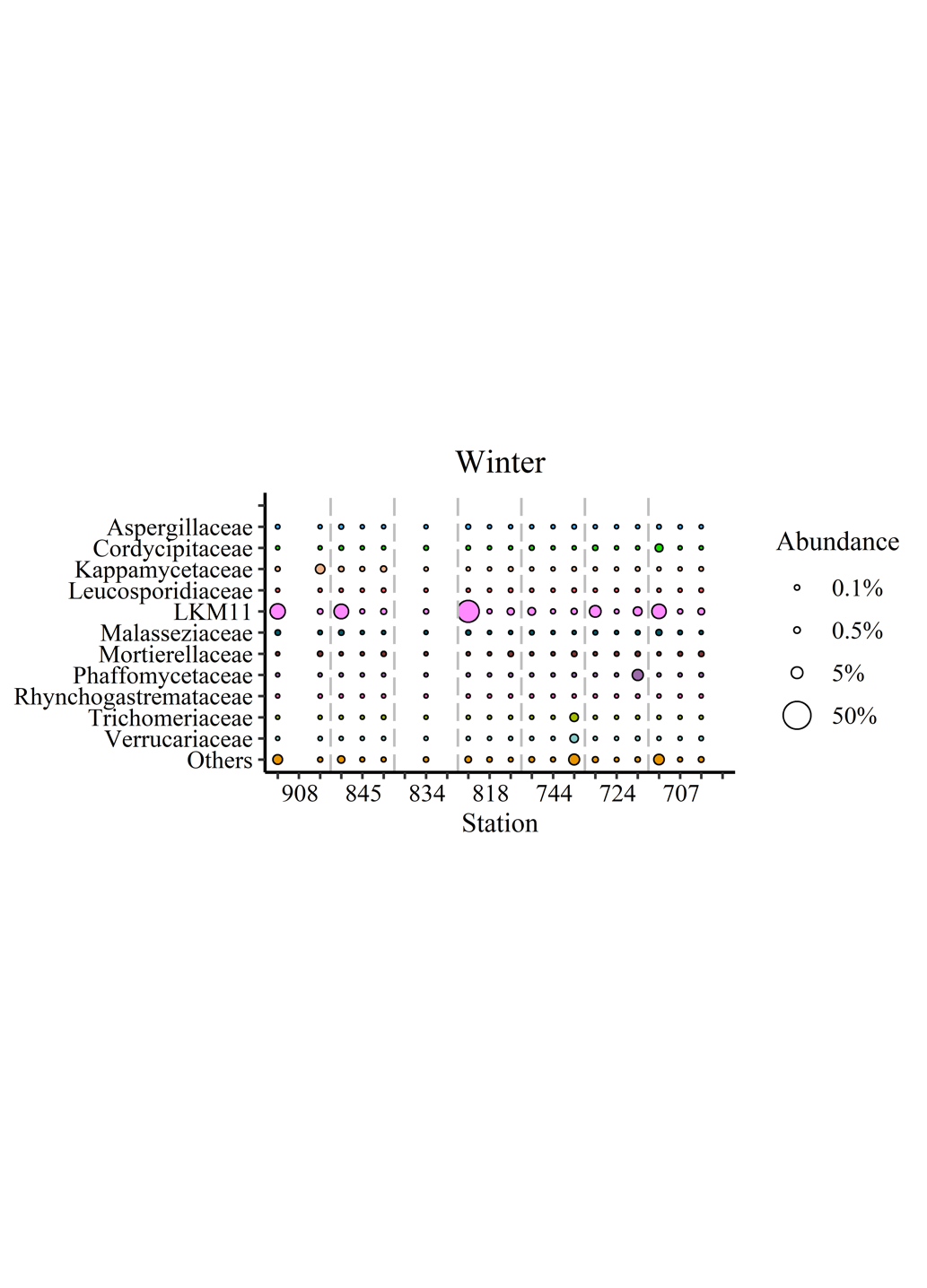

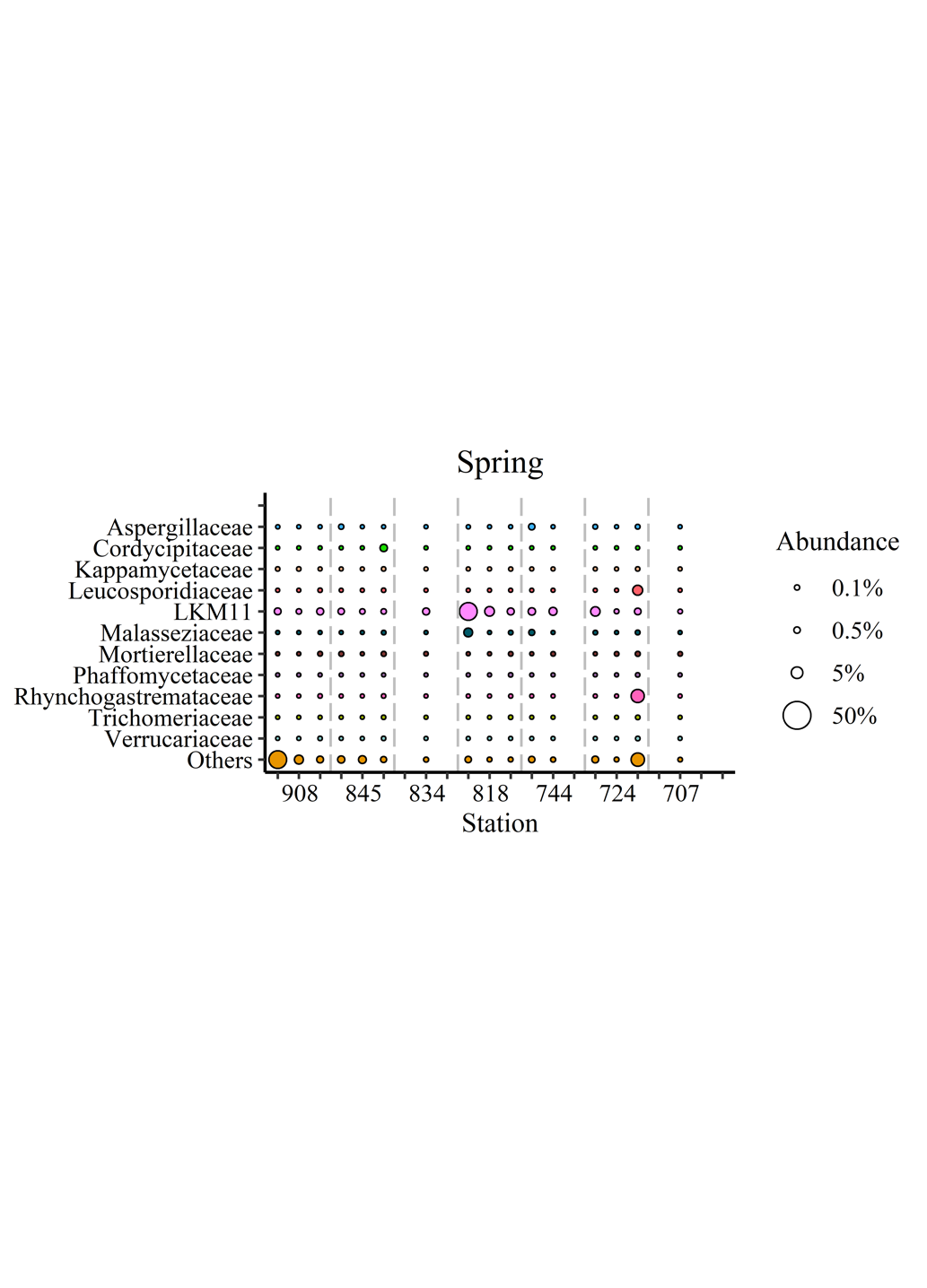

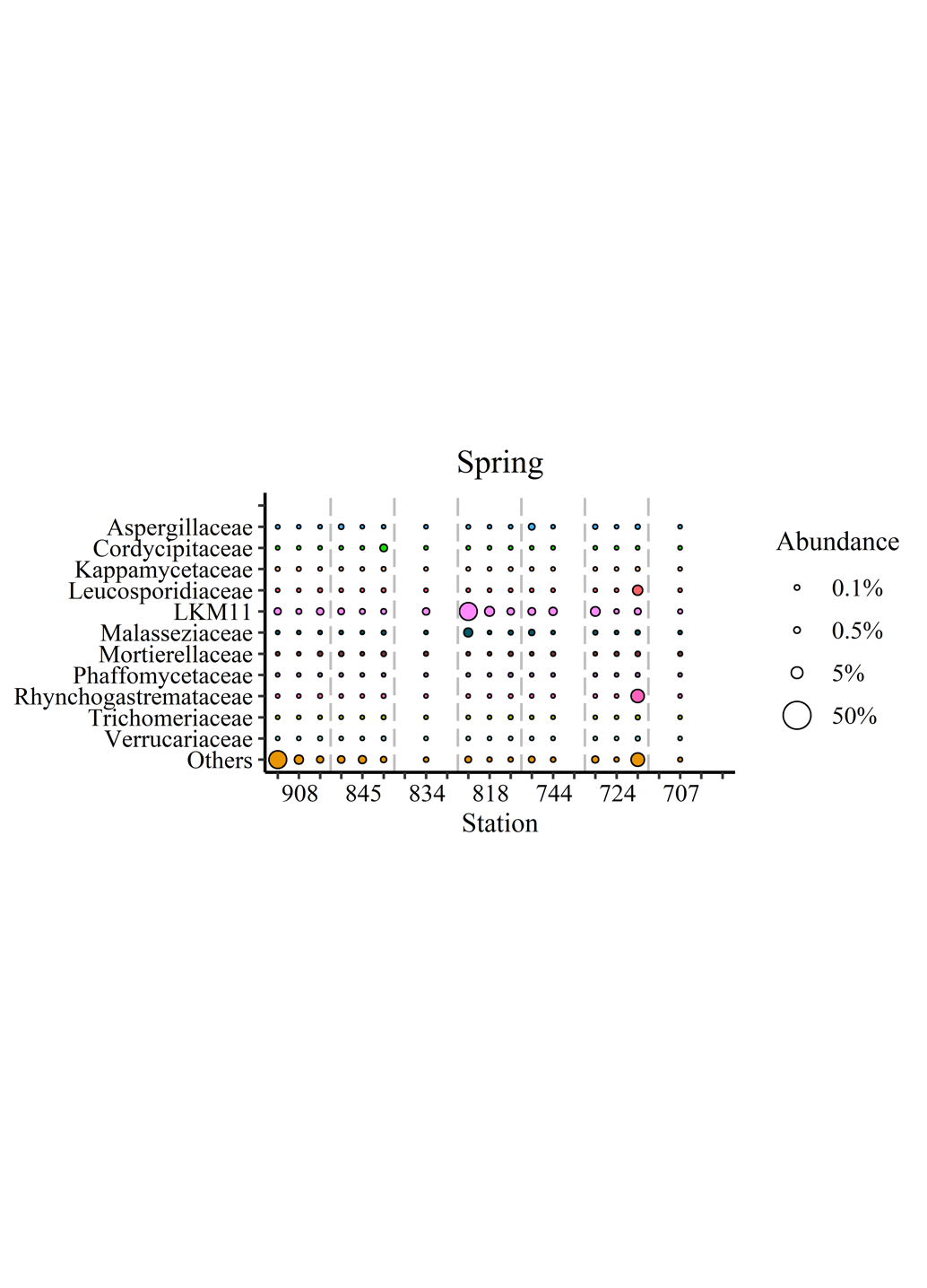


**Winter**

**Spring**

**Summer**

**Autumn**

**FIG S6.** Relative abundance of major families of Fungi in the Chesapeake Bay. Seasonal variations (winter, spring, summer and autumn) from each sampling site for three consecutive years are included (unless it is unavailable). Bubble size represents the relative abundance of major families within each sample.


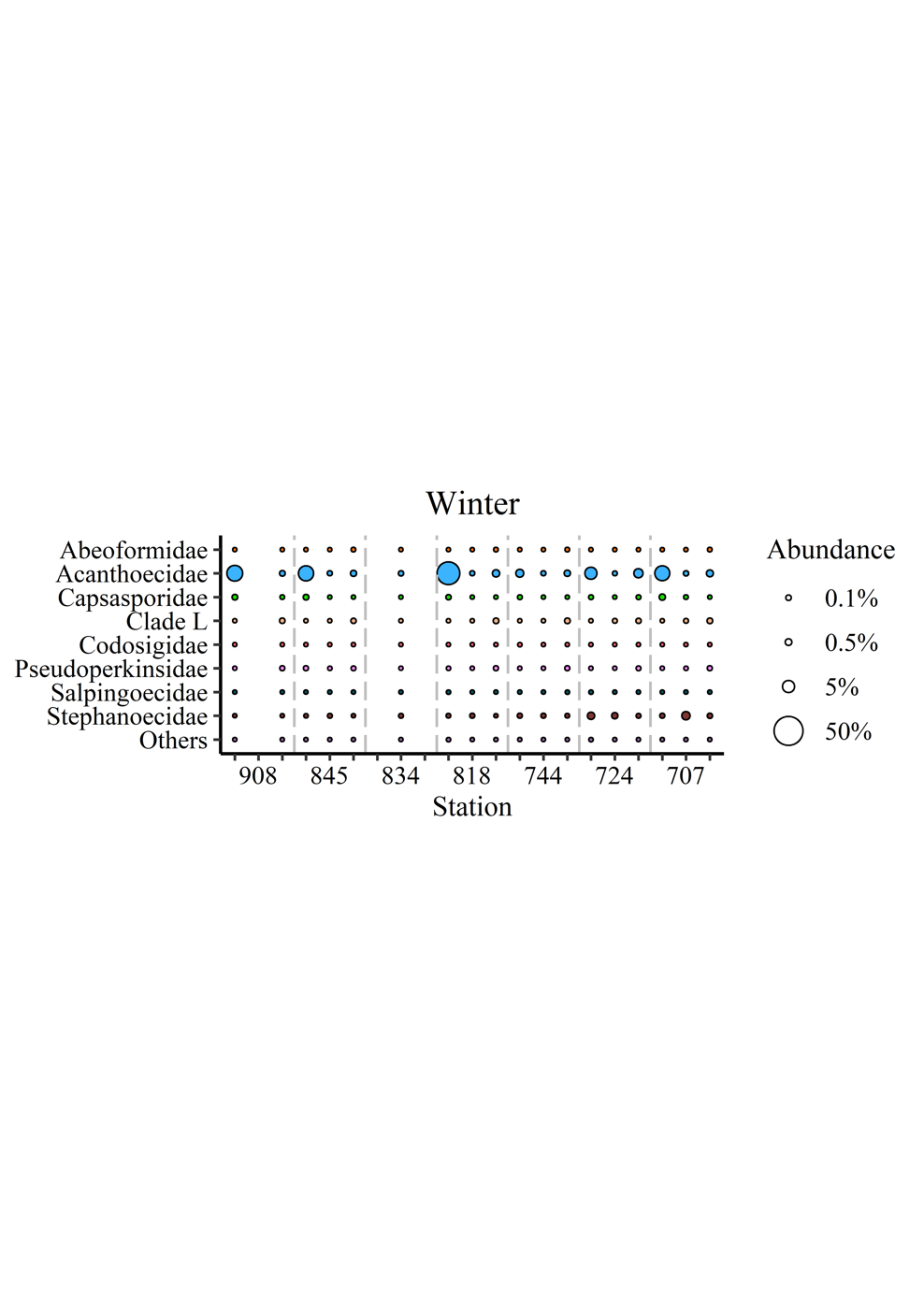

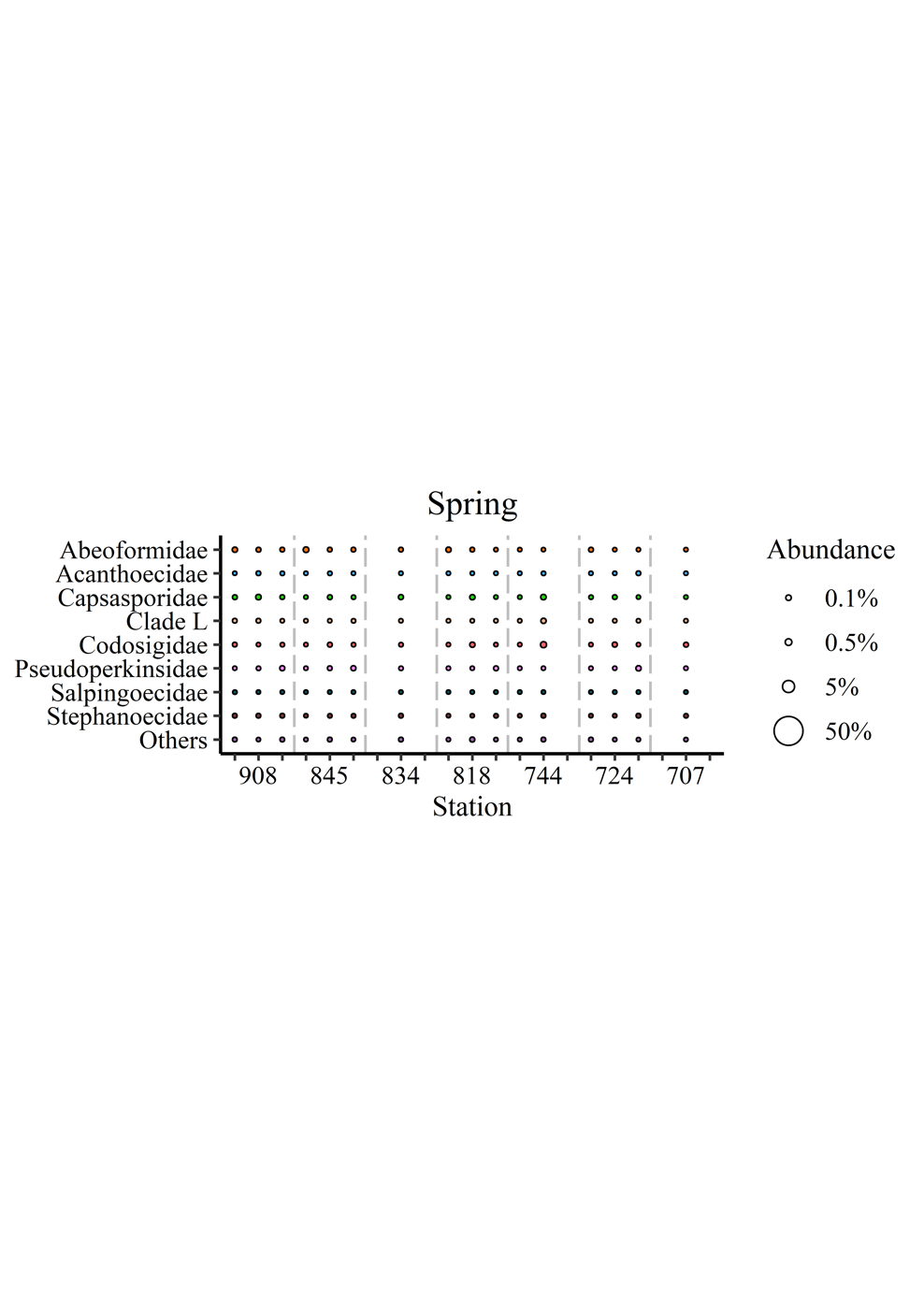

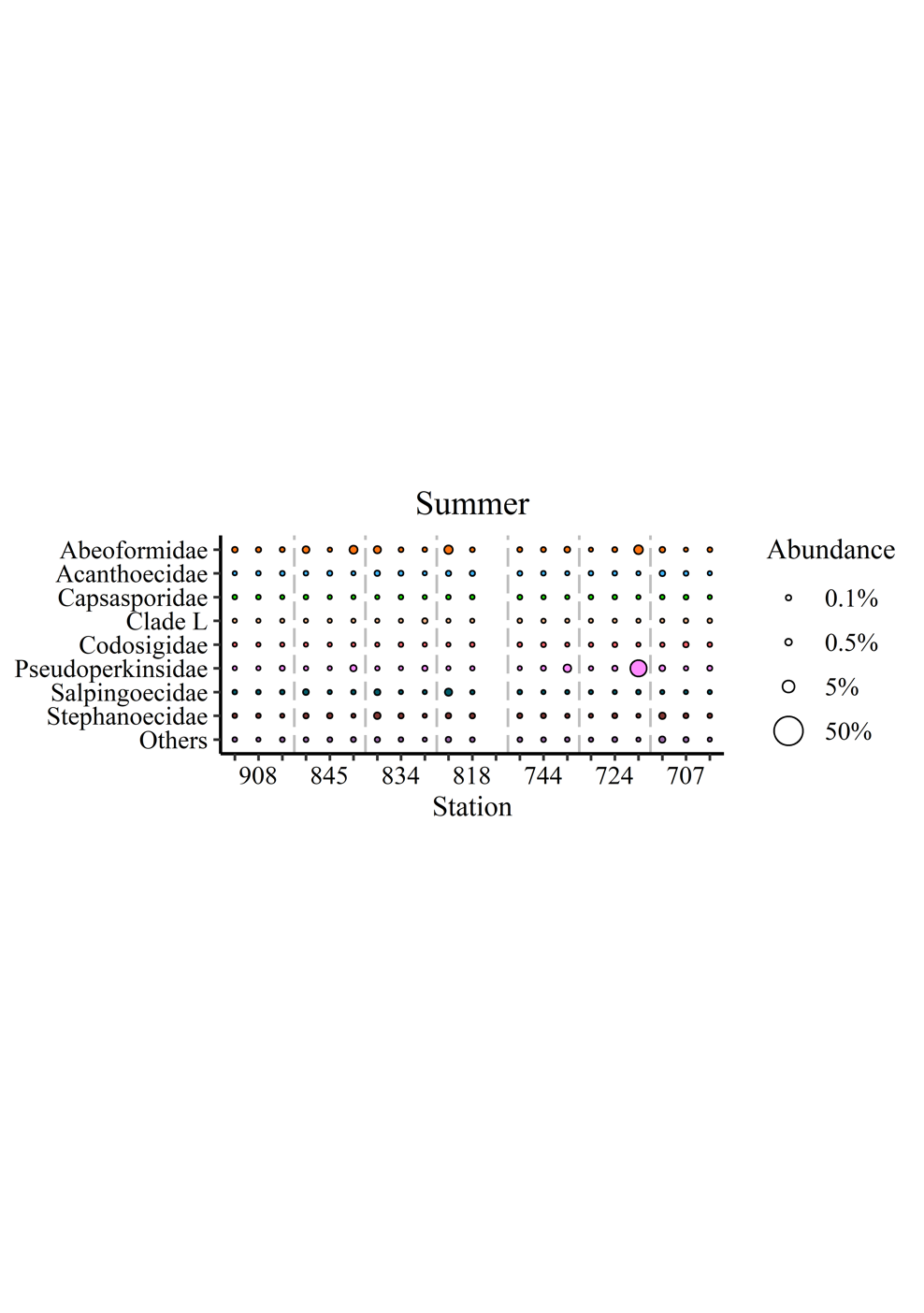

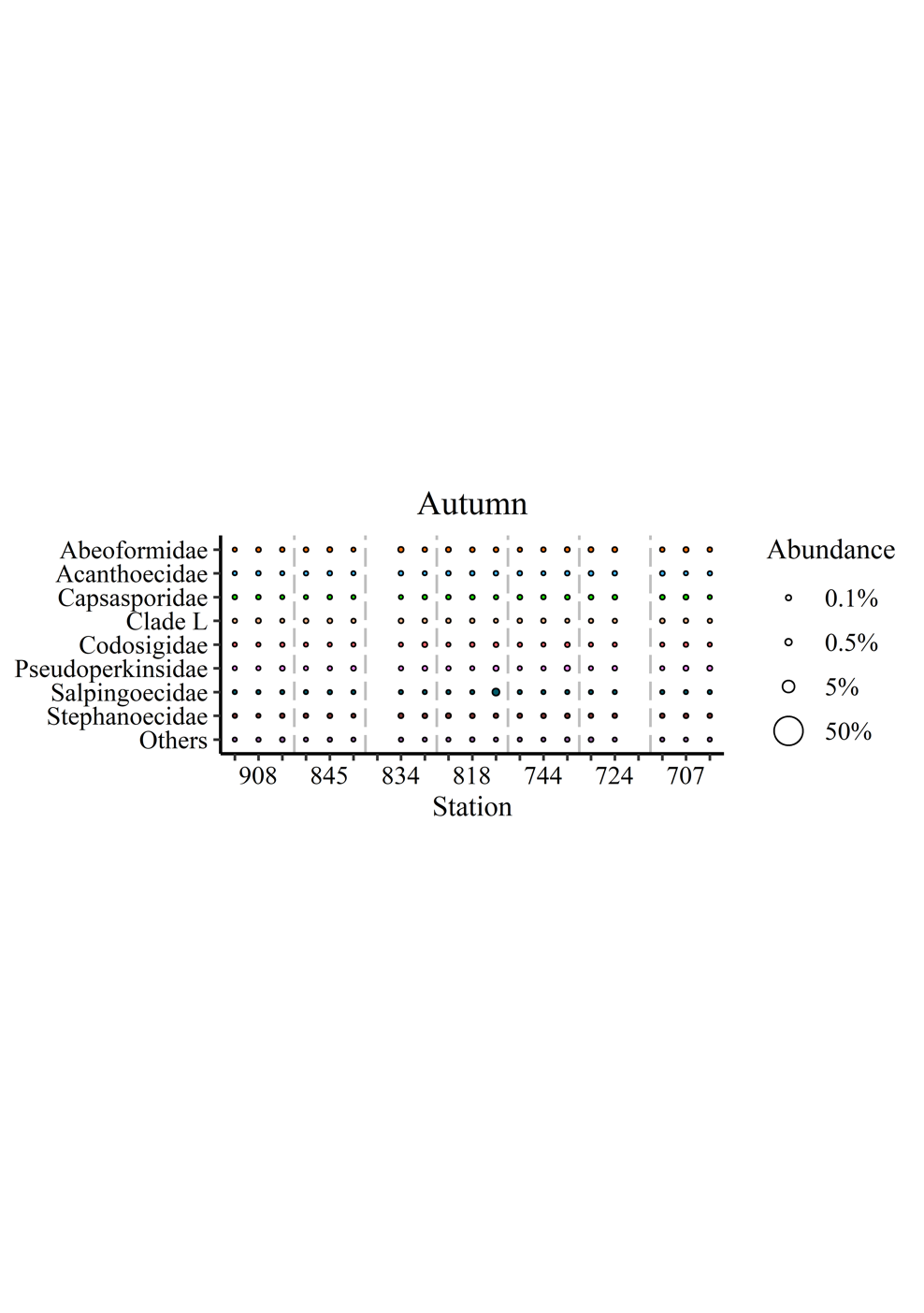

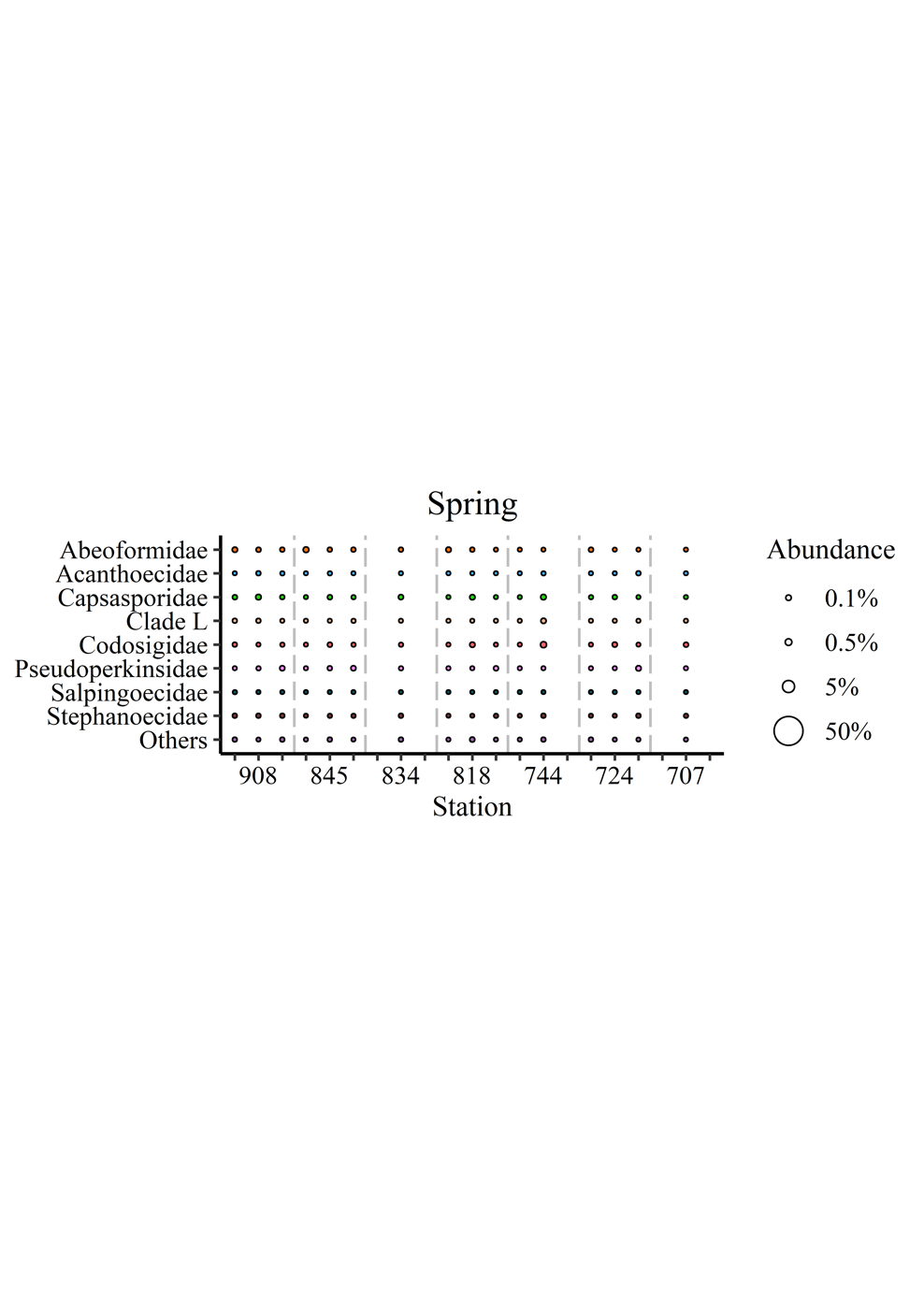


**Winter**

**Spring**

**Summer**

**Autumn**

**FIG S7.** Relative abundance of major families of Holozoa in the Chesapeake Bay. Seasonal variations (winter, spring, summer and autumn) from each sampling site for three consecutive years are included (unless it is unavailable). Bubble size represents the relative abundance of major families within each sample.


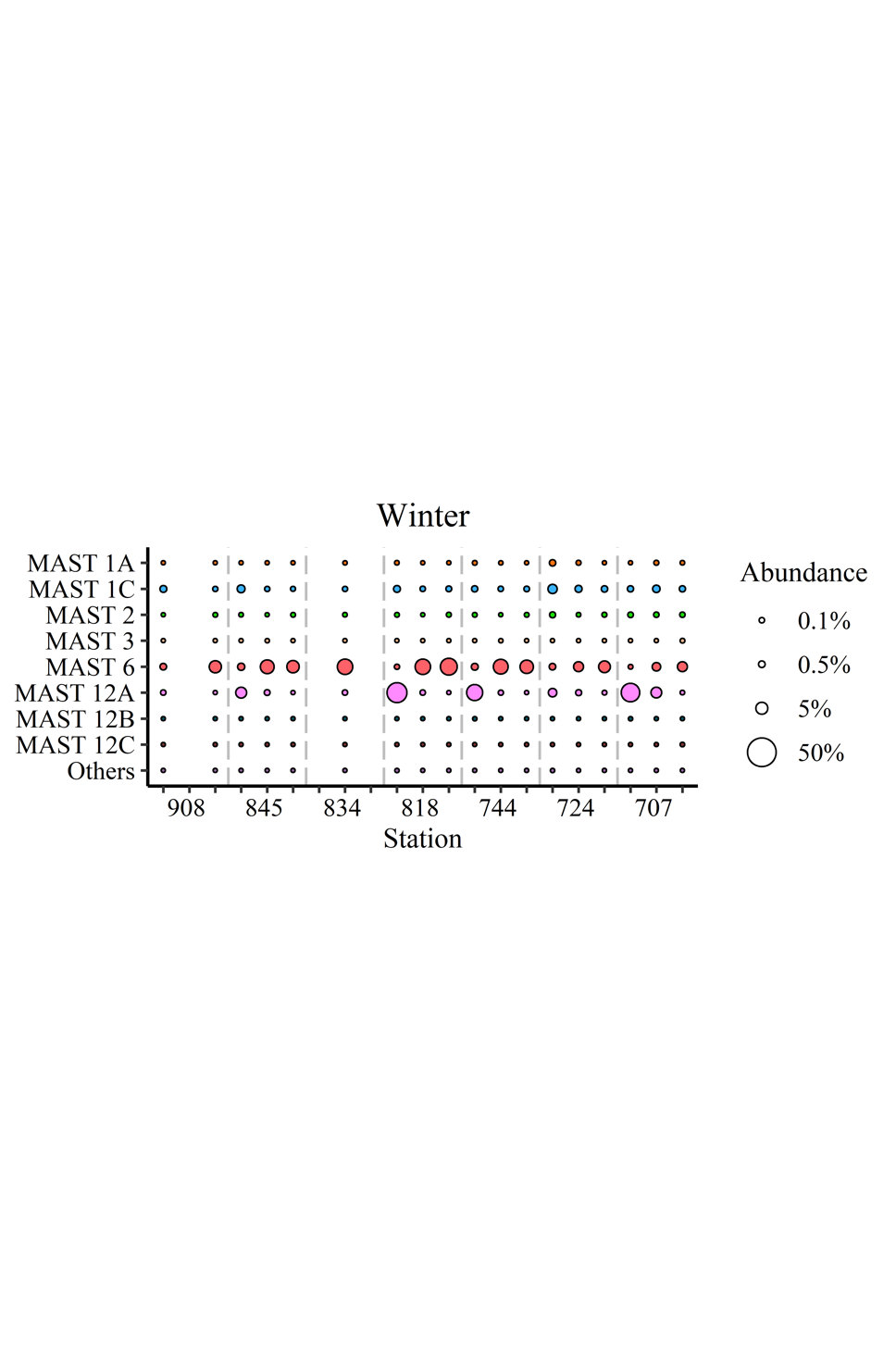

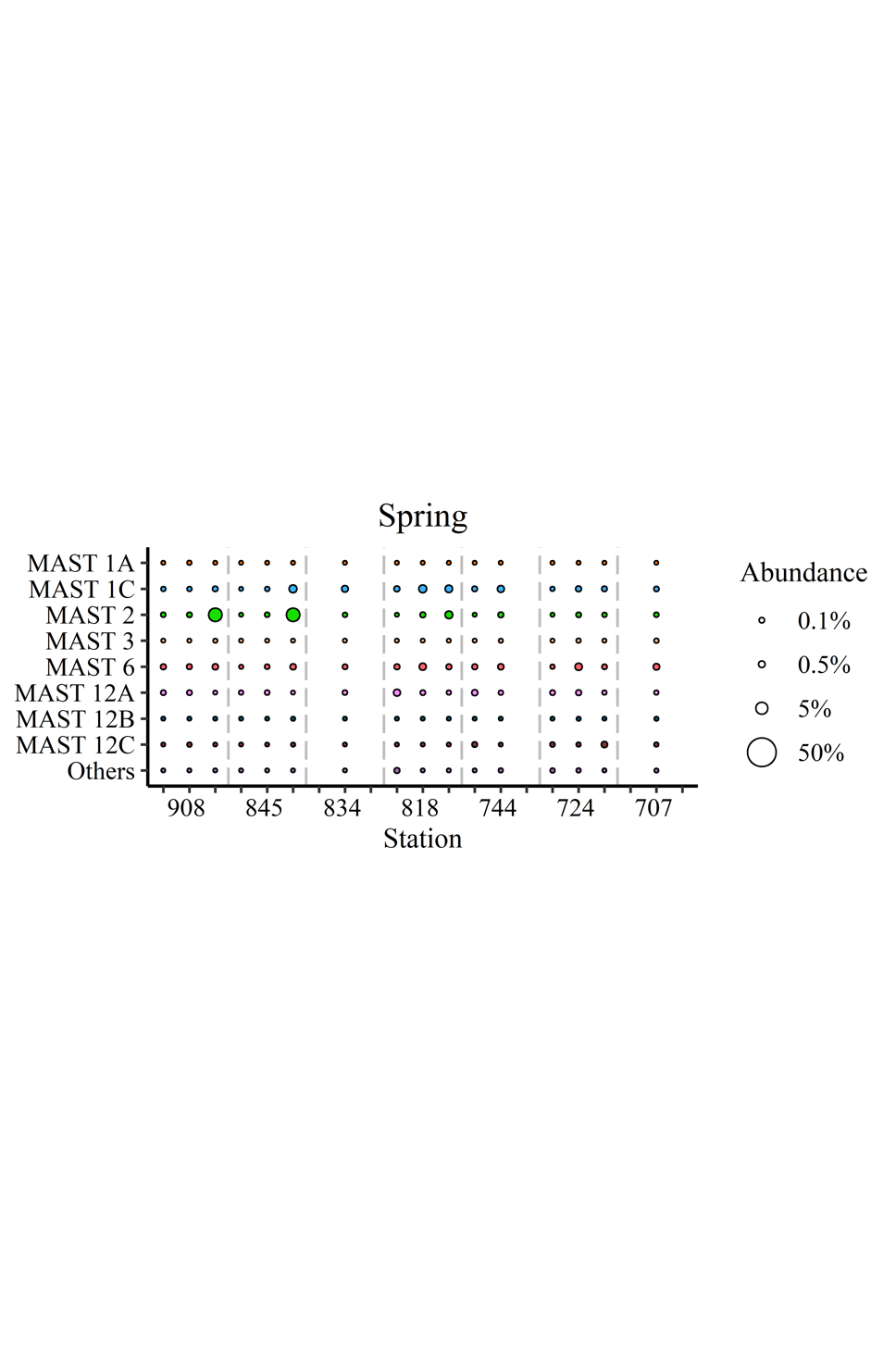

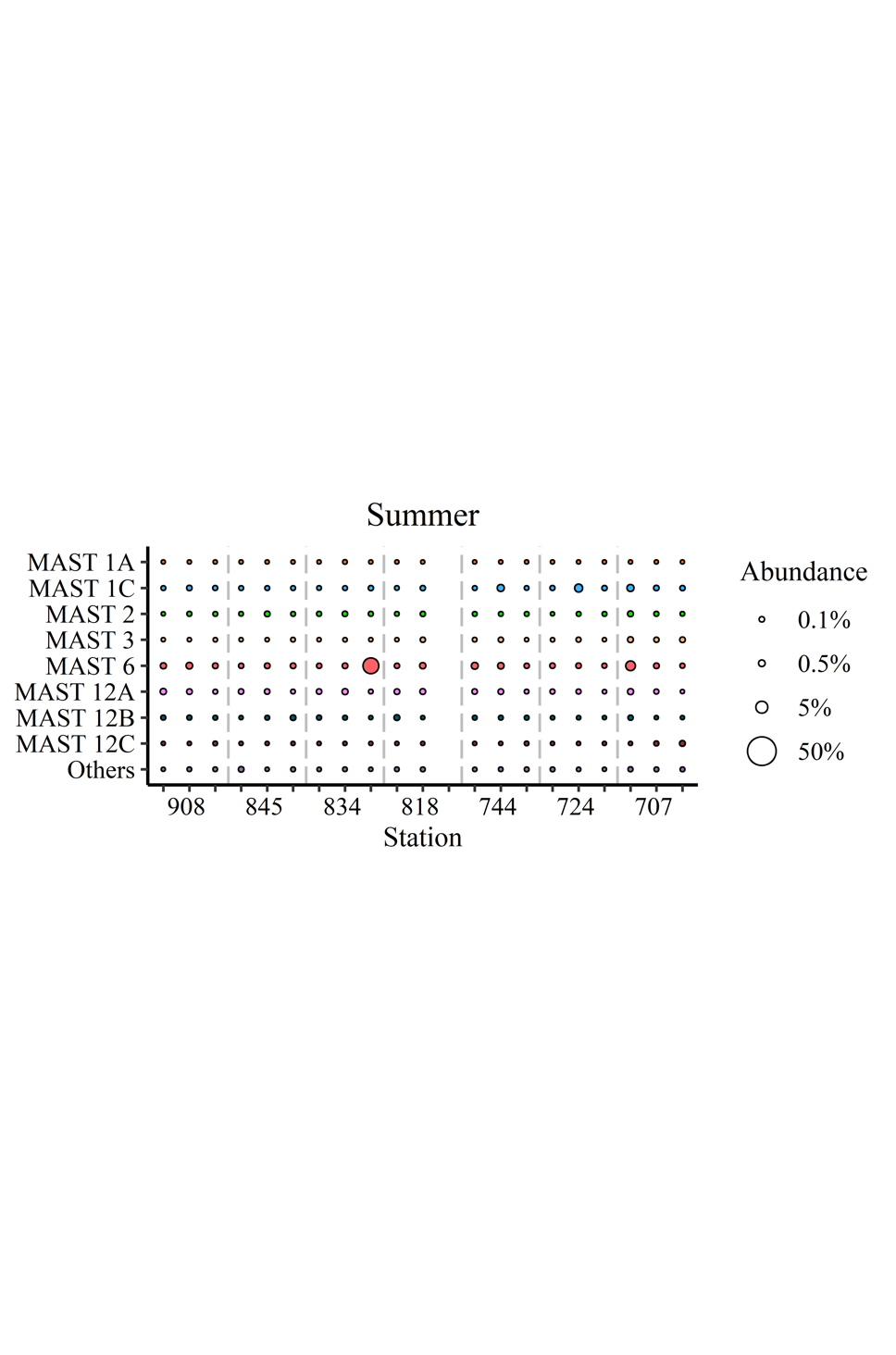

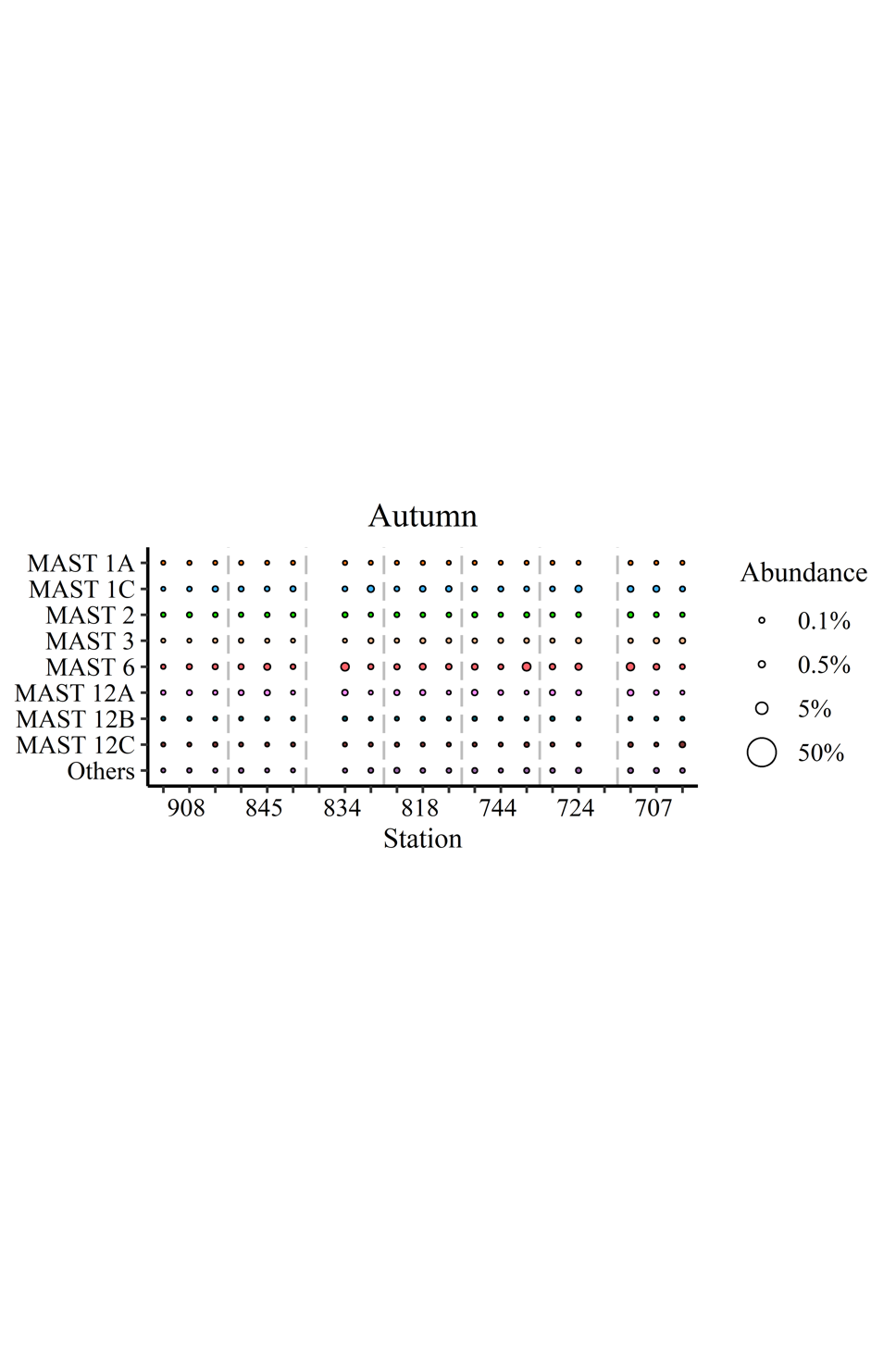

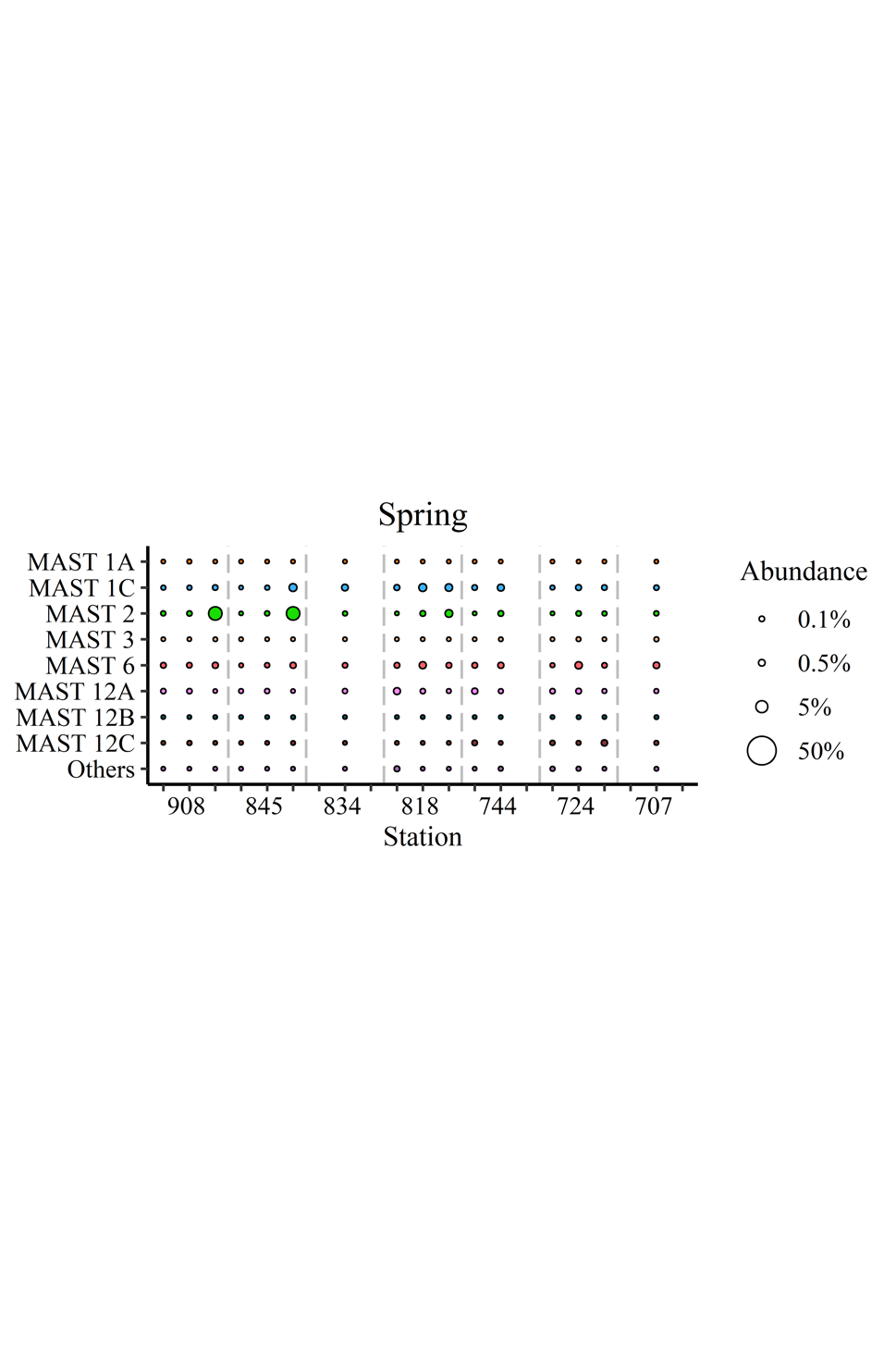


**Winter**

**Spring**

**Summer**

**Autumn**

**FIG S8.** Relative abundance of major families of MASTs in the Chesapeake Bay. Seasonal variations (winter, spring, summer and autumn) from each sampling site for three consecutive years are included (unless it is unavailable). Bubble size represents the relative abundance of major families within each sample.


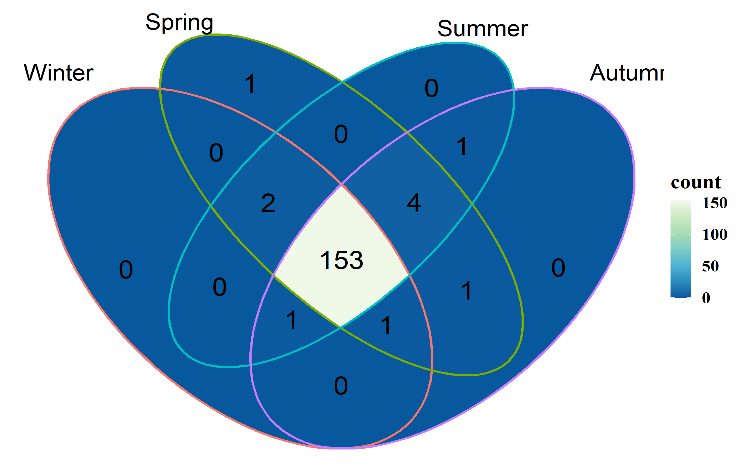

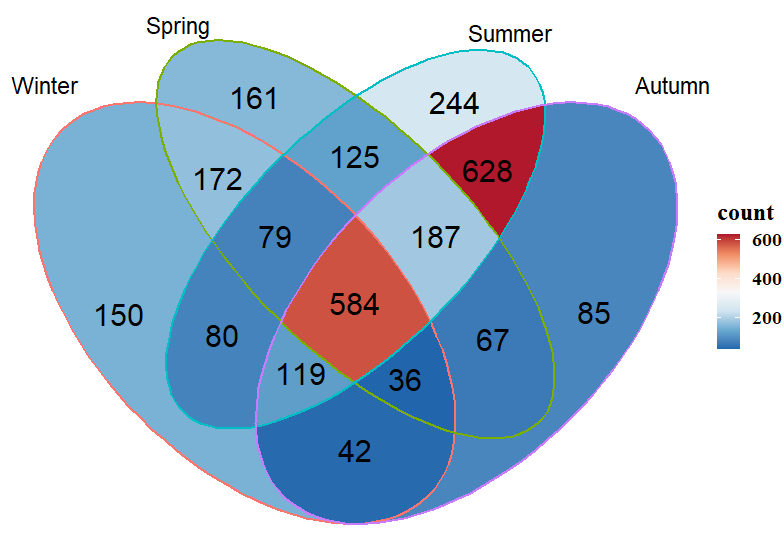

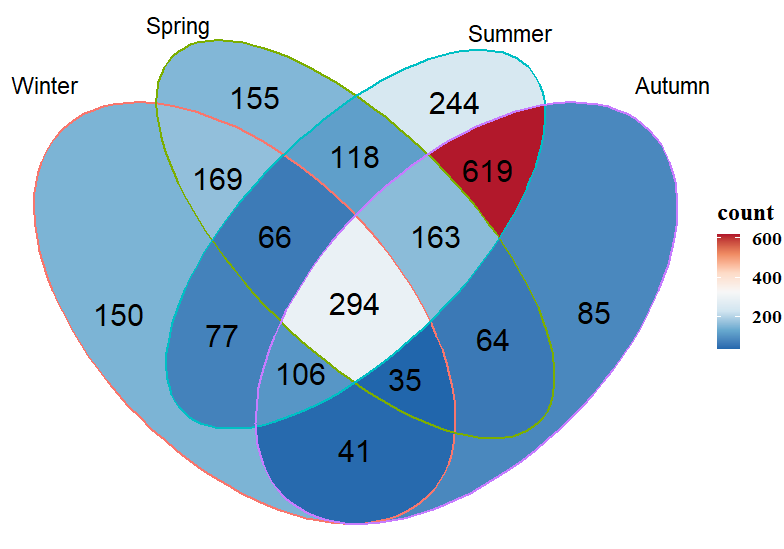


**All**

**Abundant**

**Rare**

**Winter**

**Spring**

**Summer**

**Autumn**

**Winter**

**Spring**

**Summer**

**Autumn**

**Winter**

**Spring**

**Summer**

**Autumn**


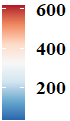


**FIG S9.** Venn diagram showing the numbers of unique and shared ASVs between groups.


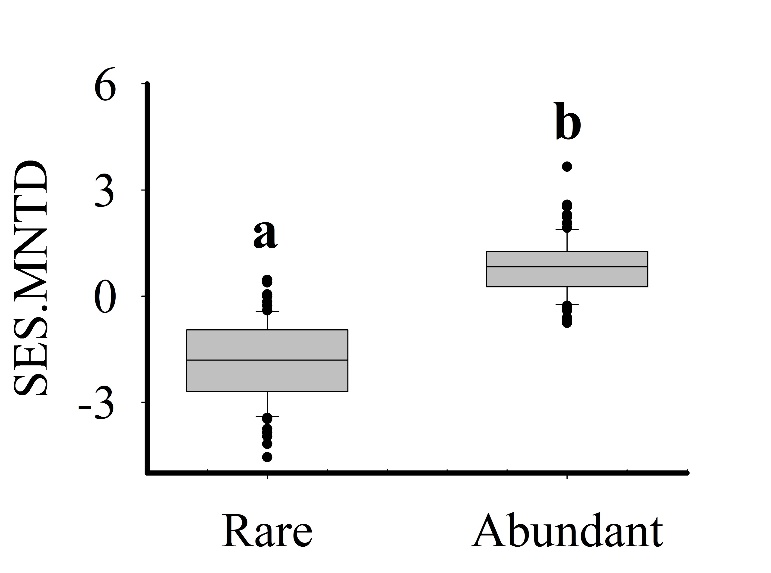

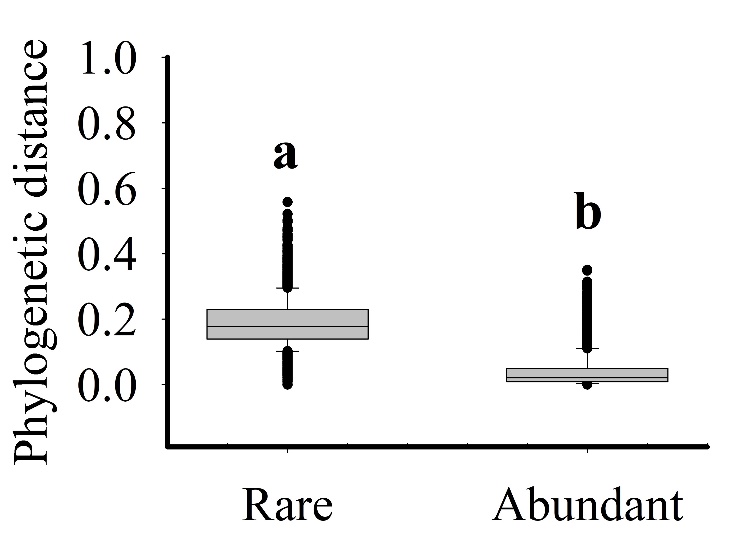


**a**

**b**

**FIG S10.** Phylogenetic distance between abundant and rare sub-communities of planktonic eukaryotes in the Bay based on SES.MNTD and βMNTD statistical analysis.

**TABLE S1.** Cell counts (cells/L) and the proportion of major microalgal groups in the Chesapeake Bay. Station names are coded with sampling station and time (month and year). For instance, 908_303 represents a sample from station 908 in March 2003.

**TABLE S2.** Comparisons of major eukaryotic groups across season and space.

**TABLE S3.** Correlations between major eukaryotic phyla and environmental factors in the Chesapeake Bay.

**TABLE S4.** Relative abundance of eukaryotic taxa in the Chesapeake Bay. Station names are coded with sampling station and time (month and year). For instance, 908_303 represents a sample from station 908 in March 2003. Potential HAB species are also identified and listed.

**TABLE S5.** Correlations between individual eukaryotic taxa and environmental factors in the Chesapeake Bay.

**TABLE S6.** Correlations between alpha diversity of eukaryotic communities with space (sampling station/salinity), season and year. Significant results are in bold.

**TABLE S7.** Spearman correlations of temperature, salinity and other environmental factors with the alpha diversity of eukaryotic communities. Significant correlations (*P*<0.05) are in bold (black: positive and red: negative correlations).

**TABLE S8.** Indicator species in different seasons. Only significant ASVs with associated statistic values greater than 0.45 are listed.

**TABLE S9.** Spearman's correlations of planktonic eukaryotes with environmental variables in the Bay based on Mantel tests. Significant results are in bold.
